# On the Bioactivity of *Echinacea purpurea* Extracts to Modulate the Production of Inflammatory Mediators

**DOI:** 10.3390/ijms232113616

**Published:** 2022-11-06

**Authors:** Sara F. Vieira, Virgínia M. F. Gonçalves, Carmen P. Llaguno, Felipe Macías, Maria Elizabeth Tiritan, Rui L. Reis, Helena Ferreira, Nuno M. Neves

**Affiliations:** 1 3B’s Research Group, I3Bs—Research Institute on Biomaterials, Biodegradables and Biomimetics, University of Minho, Headquarters of the European Institute of Excellence on Tissue Engineering and Regenerative Medicine, AvePark, Parque de Ciência e Tecnologia, Zona Industrial da Gandra, Barco, 4805-017 Guimarães, Portugal; 2 ICVS/3B’s—PT Government Associate Laboratory, 4710-057 Braga/Guimarães, Portugal; 3 TOXRUN—Toxicology Research Unit, University Institute of Health Sciences (IUCS), CESPU, CRL, 4585-116 Gandra, Portugal; 4 UNIPRO—Oral Pathology and Rehabilitation Research Unit, University Institute of Health Sciences (IUCS), CESPU, CRL, 4585-116 Gandra, Portugal; 5 Departamento de Edafoloxía e Química Agrícola, Facultade de Bioloxía, Universidade de Santiago de Compostela, 15782 Santiago de Compostela, Spain; 6 Interdisciplinary Centre of Marine and Environmental Research (CIIMAR), University of Porto, Terminal de Cruzeiros do Porto de Leixões, Avenida General Norton de Matos, S/N, 4450-208 Matosinhos, Portugal; 7 Laboratório de Química Orgânica e Farmacêutica, Departamento de Ciências Químicas, Faculdade de Farmácia da Universidade do Porto, Rua Jorge de Viterbo Ferreira 228, 4050-313 Porto, Portugal

**Keywords:** *Echinacea purpurea* extracts, inflammation, pro-inflammatory cytokines, reactive oxygen and nitrogen species, macrophages, alkylamides, phenols/acids

## Abstract

Inflammatory diseases are the focus of several clinical studies, due to limitations and serious side effects of available therapies. Plant-based drugs (e.g., salicylic acid, morphine) have become landmarks in the pharmaceutical field. Therefore, we investigated the immunomodulatory effects of flowers, leaves, and roots from *Echinacea purpurea*. Ethanolic (EE) and dichloromethanolic extracts (DE) were obtained using the Accelerated Solvent Extractor and aqueous extracts (AE) were prepared under stirring. Their chemical fingerprint was evaluated by liquid chromatography–high resolution mass spectrometry (LC-HRMS). The pro- and anti-inflammatory effects, as well as the reduction in intracellular reactive oxygen and nitrogen species (ROS/RNS), of the different extracts were evaluated using non-stimulated and lipopolysaccharide-stimulated macrophages. Interestingly, AE were able to stimulate macrophages to produce pro-inflammatory cytokines (tumor necrosis factor -TNF-α, interleukin -IL-1β, and IL-6), and to generate ROS/RNS. Conversely, under an inflammatory scenario, all extracts reduced the amount of pro-inflammatory mediators. DE, alkylamides-enriched extracts, showed the strongest anti-inflammatory activity. Moreover, *E. purpurea* extracts demonstrated generally a more robust anti-inflammatory activity than clinically used anti-inflammatory drugs (dexamethasone, diclofenac, salicylic acid, and celecoxib). Therefore, *E. purpurea* extracts may be used to develop new effective therapeutic formulations for disorders in which the immune system is either overactive or impaired.

## 1. Introduction

Inflammation is a natural and essential defense process of the organism against noxious stimuli and trauma [1]. Macrophages, a key immune cell of the first line of the host defense, are activated in the presence of several signals, for example, lipopolysaccharide (LPS), a major constituent of the outer wall of gram-negative bacteria [2]. When activated, intracellular signaling inflammatory pathways are triggered, which in turn stimulates the production and release of several inflammatory mediators to eliminate the harmful stimulus and restore the homeostasis of the body [1]. Tumor necrosis factor (TNF)-α, interleukin (IL)-1β, and IL-6 are the main pro-inflammatory cytokines released during an inflammatory process [3]. TNF-α is a pleiotropic cytokine that induces the proliferation of immune cell clones and stimulates the differentiation and recruitment of naïve immune cells [4]. IL-1β promotes the recruitment of inflammatory cells at the site of inflammation and induces the production of cyclooxygenase-2 (COX-2) and inducible nitric oxide synthase (iNOS) [5]. IL-6, being a pleiotropic cytokine, promotes the expansion and activation of T cells, the differentiation of B cells, and the regulation of the acute-phase response [6]. Besides pro-inflammatory cytokines, reactive oxygen and nitrogen species (ROS/RNS) are also rapidly produced in large amounts to effectively kill the pathogens [7,8]. Furthermore, the organism has mechanisms to control overexuberant immune responses, avoiding the damage of the own cells and tissues [9,10]. However, if a disturbance in the homeostasis of the immune response occurs, a persistent inflammatory process can be observed. Chronic inflammation can lead to serious pathological conditions, such as autoimmune, cardiovascular, and neurodegenerative diseases [11,12]. Nowadays, the control of the chronic inflammatory process is essentially regulated by non-steroidal anti-inflammatory drugs (NSAIDs; e.g., diclofenac, celecoxib, salicylic acid) [13], corticosteroids drugs (e.g., dexamethasone and betamethasone) [14], and conventional or biological disease-modifying antirheumatic drugs (DMARDs; e.g., methotrexate or anti-TNF-α and anti-IL-6 antibodies, respectively) [15]. However, these drugs have been related to severe side effects, mainly if their administration lasts for long periods [16,17,18]. Therefore, more effective and safer anti-inflammatory drugs are urgently needed.

Nature is a vast source of bioactive compounds. Particularly, plants produce several chemical compounds, known as secondary metabolites, to protect them against the surrounding environment, such as possible herbivores and pathogens or even to mitigate the effects of radiation [19]. Interestingly, many of those chemicals, such as morphine, salicylic acid, paclitaxel, and artemisinin, have been widely used to successfully treat different human diseases.

*Echinacea purpurea* is an indigenous North American purple coneflower, belonging to the Asteraceae family. Traditional preparations of *Echinacea* were used to prevent and relieve a variety of different inflammatory conditions, including swollen gums, sore throats, skin inflammation, and gastrointestinal disorders [20]. Being also considered an immune booster, nowadays, *E. purpurea* preparations are used to prevent cold and flu and to heal sore throats and respiratory infections [21]. In fact, a dual effect of *E. purpurea* extracts on immune cells has been reported. Due to their strong immunomodulatory activity, they could stimulate or suppress the immune system. For instance, *E. purpurea* extracts can promote both phenotypic and functional maturation of dendritic cells [22], and activate and polarize M1 macrophages [23]. Moreover, *E. purpurea* extracts can inhibit IL-2 production by T cells [24] and TNF-α by macrophages [25]. The immunomodulatory effects were associated with the presence of a different class of bioactive molecules, including caffeic acid derivatives, alkylamides, and polysaccharides [22,26,27,28,29].

Various extraction techniques were already employed to extract bioactive compounds from *E. purpurea*. Classical extraction, such as solvent extraction with or without stirring, infusions, decoctions, maceration, and soxhlet [30,31,32,33,34,35], uses high temperatures to obtain high yields of the bioactive compounds [36]. However, it is well known that temperature can denature several compounds, reducing their biological activity. Despite the use of ultrasounds [37] and microwaves [30] present many advantages in comparison with the classical methods (e.g., less extraction time and solvent consumption and higher yield), they are also associated with degradation and loss of integrity of bioactive compounds, due to the production of radicals [37]. Besides the technique, the solvent used in the extraction also affects drastically the amount and type of bioactive compounds extracted. Ethanol, hydroethanol, methanol, and chloroform have been reported for the extraction of bioactive compounds for *E. purpurea* [30,31,32,33,35,38]. Moreover, to the best of our knowledge, only two reports used dichloromethane in the extraction process, but do not refer in vitro studies with immune cells [34,39].

Considering the importance of the combination of the extraction technique and the solvent to obtain the desired bioactive compounds, herein, the Accelerated Solvent Extraction (ASE) technique was selected to prepare ethanolic extracts (EE) and dichloromethanolic extracts (DE) obtained from flowers (F), leaves (L), and roots (R). The innovative ASE does not compromise the extract bioactivity since it allows to increase the extraction yield and to reduce the time of extraction at low temperatures. The aqueous extracts (AE) were produced by stirring at room temperature (RT). The extraction yield was calculated and the nine *E. purpurea* extracts were characterized based on their fingerprint of bioactive compounds by liquid chromatography–high resolution mass spectrometry (LC-HRMS). Two different approaches were designed to evaluate their immunostimulatory and immunosuppressive activities. Their capacity to increase or decrease, respectively, the concentration of a panel of pro-inflammatory cytokines, namely IL-6, IL-1β, and TNF-α, as well as the intracellular ROS/RNS generation were investigated, using non-stimulated or LPS-stimulated macrophages. In order to mimic in a simple way, the sequence of events developing in an inflammatory clinical condition, in this experimental model, a pro-inflammatory state (macrophages exposed to LPS) was firstly induced, and then the plant extracts were added. Indeed, the plant extracts may, for instance, detrimentally affect the LPS/TLR4 signalling if added as a pretreatment [40,41]. The metabolic activity, DNA concentration, and total protein content were analyzed. The biological activities were related to the presence of different compounds. To the best of our knowledge, this is the first study that exhaustively demonstrates the efficiency of several *E. purpurea* extracts to increase or reduce free radical generation and inflammation. Additionally, it presents the most comprehensive list of phenols/acids and alkylamides of several *E. purpurea* extracts ever reported to date.

## 2. Results

### 2.1. Extraction Yield

**Figure 1:**
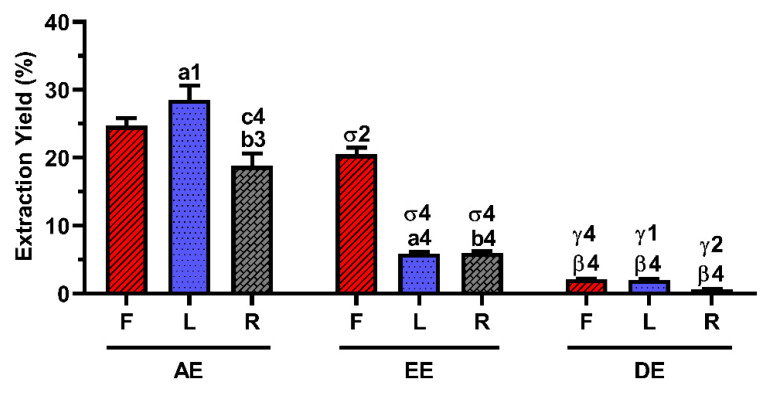
Extraction yield (%) of the different *E. purpurea* extracts. Statistically significant differences are 1 (*p* < 0.0155), 2 (*p* < 0.0088), 3 (*p* < 0.0010), and 4 (*p* < 0.0001) in comparison with a (Flowers vs. Leaves), b (Flowers vs. Roots), c (Leaves vs. Roots), σ (AE vs. EE), β (AE vs. DE), and γ (EE vs. DE). F: flowers; L: leaves; R: roots; AE: aqueous extracts; EE: ethanolic extracts; DE: dichloromethanolic extracts.

The extraction yield of each *E. purpurea* extract is presented in Figure 1. The extraction performed with water provided significantly higher extraction yields than the extraction with ethanol (EtOH) and dichloromethane (DCM), for all the plant organs studied. Using water as a solvent, the leaves (L) were the organ of the plant that significantly provided a higher amount of extract (28.5 ± 2.1%), followed by flowers (F, 24.7 ± 1.1%) and roots (R, 18.8 ± 1.8%). When the extraction was performed with EtOH, the flowers significantly gave a higher extraction yield (20.5 ± 1.0%), followed by roots (6.0 ± 0.3%) and leaves (5.9 ± 0.3%). However, no significant differences were observed in the yield obtained between flowers (2.2 ± 0.1%), leaves (2.1 ± 0.1%), or roots (0.7 ± 0.1%) when DCM was used. Comparing all the *E. purpurea* extracts, AE-L gave the highest extraction yield, followed by AE-F, EE-F, AE-R, EE-R ≈ EE-L, DE-F ≈ DE-L, and DE-R.

### 2.2. Composition of the E. purpurea Extracts

The LC-HRMS technique allowed unequivocal identification of the bioactive compounds present in *E. purpurea* extracts. Table 1 presents the identified phenolic/acidic compounds and alkylamides in each *E. purpurea* extract. Both product ion and relative intensities for fragments of the standards perfectly matched those obtained for the compounds present in *E. purpurea* extracts (represented by smooth grey shaded in Table 1 and Supplementary Tables S1 and S2). The retention times (*t_R_*), the precursor ions, and the ion products are listed in Supplementary Table S2 for phenols/acids and Supplementary Table S3 for alkylamides. Thirteen different phenols/acids and thirty different alkylamides were identified in the *E. purpurea* extracts. Each extract exhibited different patterns of phenols/acids and alkylamides.

EtOH extracted phenolic/acidic compounds more efficiently than water and DCM. For EE, the flowers presented the highest number of identified phenols/acids (11), followed by roots (8) and leaves (7). For AE, the same number of phenols/acids (7) was identified in flowers and leaves, while in the roots, only 3 phenols/acids were identified. In DE, 5 phenols/acids were identified in leaves, followed by roots (4) and flowers (3).

DCM and EtOH had an increased capability to extract alkylamides, compared to water. In the three tested solvents, the alkylamides were more pronounced in roots and flowers than leaves. For DE, 24 and 20 alkylamides were identified in roots and flowers, respectively. A similar result was observed for EE (23 and 19 compounds identified in roots and flowers, respectively). In AE, 16 and 14 alkylamides were identified in roots and flowers, respectively. The leaves showed a minimum amount of alkylamides, being identified with 8 alkylamides in EE and DE and none in AE. 

Analyzing all the *E. purpurea* extracts, EE-F exhibited the highest number of phenols/acids (11), followed by EE-R (8); EE-L (7), AE-F (7) and AE-L (7); DE-L (5); DE-R (4); and DE-F (3), and AE-R (3). Regarding the alkylamides, DE-R presented the highest amount of alkylamides (24), followed by EE-R (23); DE-F (20); EE-F (19); AE-R (16); AE-F (14); DE-L (8) and EE-L (8); and AE-L (0).

### 2.3. Cytotoxicity of the E. purpurea Extracts

#### 2.3.1. Non-Stimulated Macrophages

**Figure 2:**
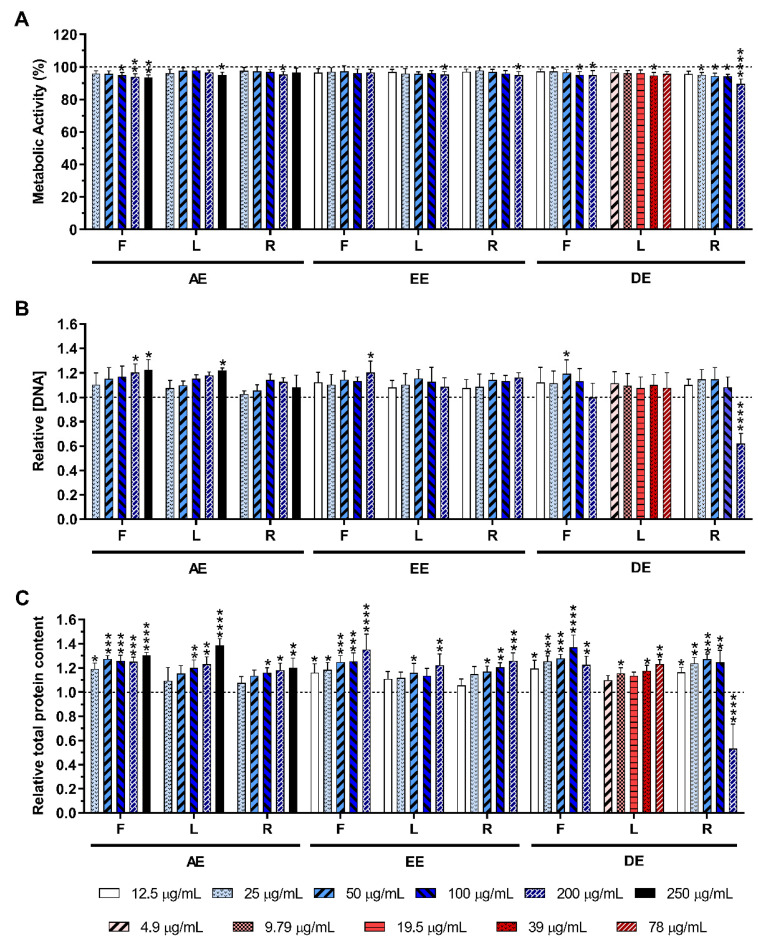
Metabolic activity (**A**), relative DNA concentration (**B**) and relative total protein content (**C**) of non-stimulated macrophages cultured in the presence of different concentrations of the *E. purpurea* extracts for 24 h of culture. The dotted line represents the metabolic activity, DNA concentration, and total protein content of negative control (non-stimulated macrophages without treatment). Statistically significant differences are * (*p* < 0.0476), ** (*p* < 0.0096), *** (*p* < 0.0010), and **** (*p* < 0.0001) in comparison to the negative control (non-stimulated macrophages without treatment) for each different tested extract. F: flowers; L: leaves; R: roots; AE: aqueous extracts; EE: ethanolic extracts; DE: dichloromethanolic extracts.

The metabolic activity, the relative DNA, and the total protein concentrations of non-stimulated macrophages in the absence or presence of the *E. purpurea* extracts at different concentrations are shown in Figure 2. As can be observed in Figure 2A, the cell metabolic activity was only significantly affected in the presence of the highest tested concentration (200 μg/mL) of DE-R. Similar behavior was observed for the DNA content (Figure 2B) and protein production (Figure 2C), where a significant decrease was noticed only in the presence of DE-R in the highest concentration (200 μg/mL). Optical micrographs of non-stimulated macrophages also confirmed that the morphology was not affected by the different *E. purpurea* extracts or the anti-inflammatory drugs used in this work, except in the presence of DE-R in the highest tested concentration (200 μg/mL) (Supplementary Figures S1–S4). Samples presented a macrophage phenotype similar to the negative control (0 μg/mL), but it was drastically affected in the presence of DE-R in the highest concentration (200 μg/mL) (Supplementary Figure S4). Indeed, after 24 h, a lower number of macrophages were attached to the bottom of the plate and their morphology became more rounded.

#### 2.3.2. LPS-Stimulated Macrophages

**Figure 3:**
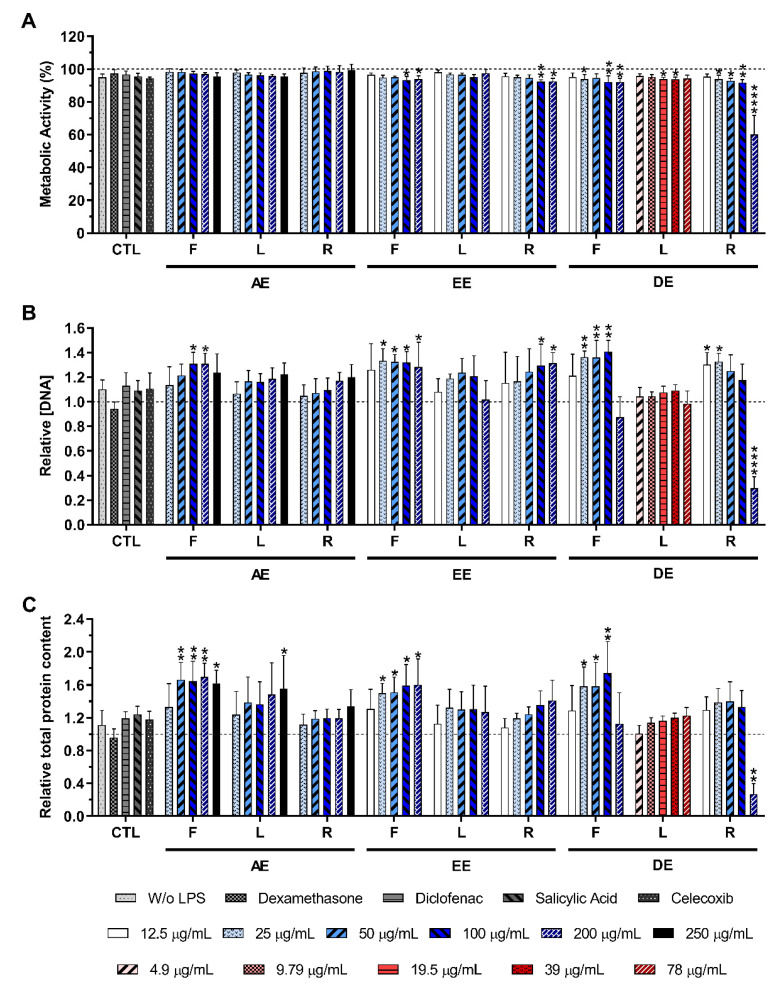
Metabolic activity (**A**), relative DNA concentration (**B**), and relative total protein content (**C**) of LPS-stimulated macrophages cultured in the presence of different concentrations of the *E. purpurea* extracts and clinically used anti-inflammatory drugs (dexamethasone, diclofenac, salicylic acid, and celecoxib) for 24 h of culture. The dotted line represents the metabolic activity, DNA concentration, and total protein content of positive control (LPS-stimulated macrophages without treatment). Statistically significant differences are * (*p* < 0.0481), ** (*p* < 0.0079), **** (*p* < 0.0001) in comparison to the positive control (LPS-stimulated macrophages without treatment) for each different tested extract. CTL: control; F: flowers; L: leaves; R: roots; AE: aqueous extracts; EE: ethanolic extracts; DE: dichloromethanolic extracts.

Figure 3 illustrates the metabolic activity, as well as the relative DNA and the protein concentration, obtained for LPS-stimulated macrophages in the absence or presence of the *E. purpurea* extracts at different concentrations. As observed for non-stimulated macrophages, the cell metabolic activity, the DNA concentration, and the total protein content were not negatively affected by *E. purpurea* extracts at different concentrations, in comparison with the positive control (LPS-stimulated macrophages without treatment), except for DE-R in the highest tested concentration (200 μg/mL) (Figure 3A–C). Optical micrographs of LPS-stimulated macrophages also confirmed the cytocompatibility of the extracts (Supplementary Figures S5–S7). Except for DE-R in the highest concentration (200 μg/mL), the tested conditions showed a macrophage like-phenotype similar to the negative control (without stimulation and extracts addition).

### 2.4. Effect of E. purpurea Extracts on Cytokine Production

#### 2.4.1. Non-Stimulated Macrophages

The pro-inflammatory activity of *E. purpurea* extracts was evaluated by assessing the levels of pro-inflammatory cytokines (IL-6, IL-1β, and TNF-α) produced by non-LPS stimulated macrophages in the cell culture medium. Non-stimulated macrophages (negative control) produced basal amounts of IL-1β (5.3 ± 1.6 arb. unit) and TNF-α (1.4 ± 1.1 arb. unit), but they did not produce measurable amounts of IL-6.

**Figure 4:**
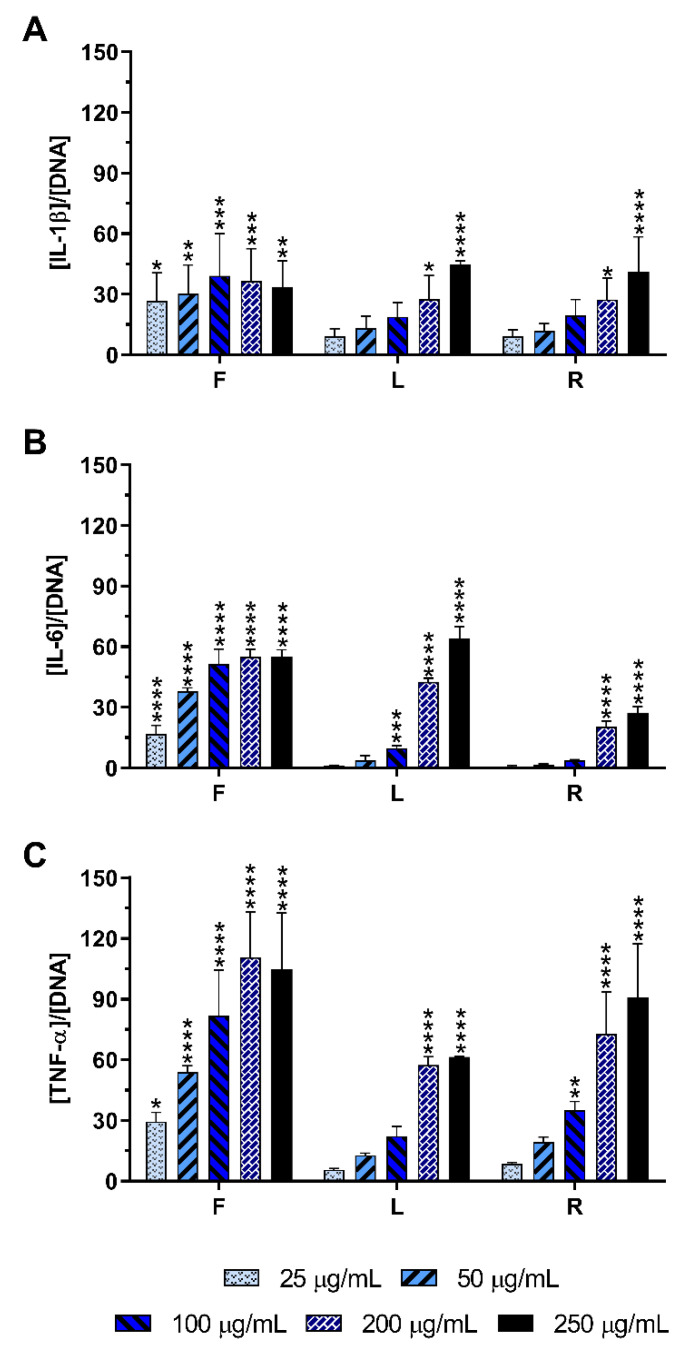
IL-1β (**A**), IL-6 (**B**), and TNF-α (**C**) production by non-stimulated macrophages cultured in the presence of different concentrations of the AE obtained from *E. purpurea* for 24 h of culture. Statistically significant differences are * (*p* < 0.0376), ** (*p* < 0.0075), *** (*p* < 0.0006), and **** (*p* < 0.0001) in comparison to the negative control (non-stimulated macrophages without treatment) for each different tested extract. F: flowers; L: leaves; R: roots.

When macrophages were incubated with the AE, an increase in those cytokines in the culture medium was observed (Figure 4), demonstrating its potential to stimulate naïve macrophages. All tested concentrations of AE-F efficiently stimulated the production of IL-1β (Figure 4A), IL-6 (Figure 4B), and TNF-α (Figure 4C). AE-L and AE-R were also able to significantly stimulate these cytokines production, although at concentrations higher than 100 µg/mL. In general, AE-F showed the greatest pro-inflammatory activity, followed by AE-R and AE-L, which presented an equivalent stimulatory activity.

The production of those pro-inflammatory cytokines by non-stimulated macrophages incubated in the presence of EE and DE were generally similar to the basal levels (Supplementary Figure S8). Only EE-R in the highest tested concentration (200 μg/mL) stimulated macrophages to produce a significant amount of TNF-α (Supplementary Figure S8C). At the same concentration, its efficacy was two times lower than those obtained for AE-F, reaching the bioactivity of the AE-L.

#### 2.4.2. LPS-Stimulated Macrophages

**Figure 5:**
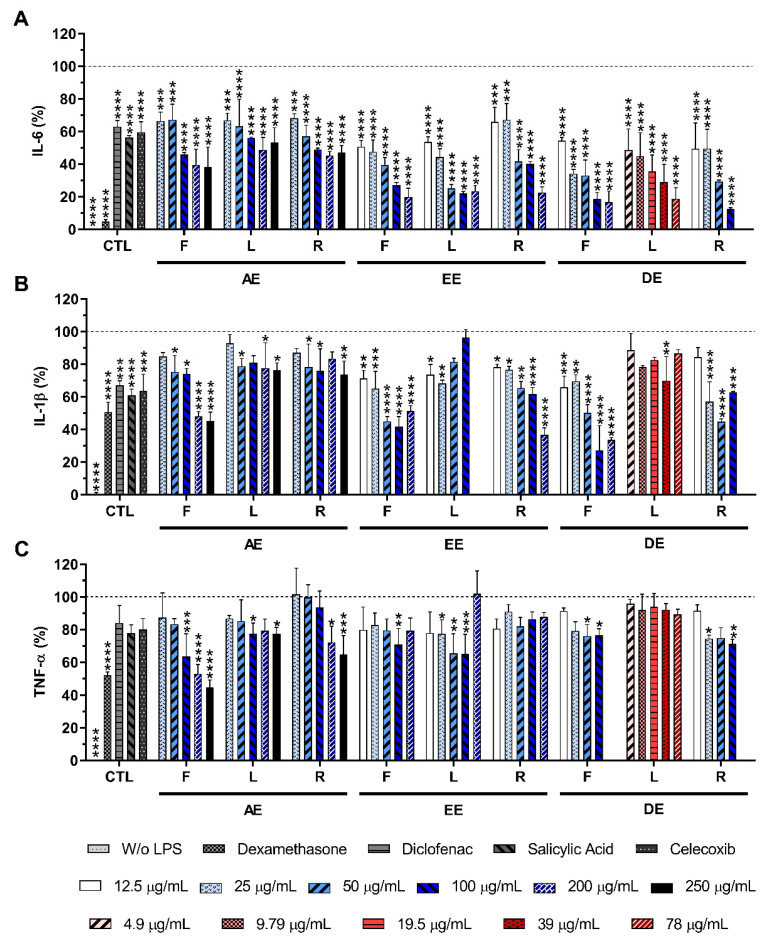
IL-6 (**A**), IL-1β (**B**), and TNF-α (**C**) percentages obtained in the presence of LPS-stimulated macrophages cultured in the presence of different concentrations of the *E. purpurea* extracts and clinically used anti-inflammatory drugs (dexamethasone, diclofenac, salicylic acid, and celecoxib, 10 μM) for 24 h of culture. The dotted line represents the maximum levels of cytokines’ production for the positive control (LPS-stimulated macrophages without treatment). Statistically significant differences are * (*p* < 0.0492), ** (*p* < 0.0090), *** (*p* < 0.0010), **** (*p* < 0.0001) in comparison to the positive control (LPS-stimulated macrophages without treatment) for each different tested extracted. CTL: control; F: flowers; L: leaves; R: roots; AE: aqueous extracts; EE: ethanolic extracts; DE: dichloromethanolic extracts.

The anti-inflammatory activity of *E. purpurea* extracts was evaluated by assessing the amount of pro-inflammatory cytokines produced by LPS-stimulated macrophages in the culture medium. The stimulation of macrophages with LPS led to a significant production of the studied pro-inflammatory cytokines, namely IL-6, IL-1β, and TNF-α (Figure 5).

Diclofenac (10 μM), celecoxib (10 μM), and salicylic acid (10 μM) led to a statistically significant reduction in the IL-6 production in 37.0 ± 3.8%, 40.3 ± 6.3%, and 43.7 ± 1.1%, respectively. Dexamethasone (10 μM) decreased the IL-6 production by 93.5 ± 1.4%, being the most efficient positive control (Figure 5A). In the presence of all *E. purpurea* extracts, IL-6 production was also drastically reduced in a concentration-dependent manner (Figure 5A). The extraction performed with DCM led to *E. purpurea* extracts with an excellent ability to reduce the IL-6 production under an inflammatory scenario, followed by EtOH and water. EE showed an activity approximated 1.3 times lower than DE. Likewise, AE exhibited an activity approximated 2 times lower than EE and approximated 2.7 times lower than DE. In all studied solvents, the extracts obtained from leaves and roots demonstrated an improved reduction in the IL-6 production, followed by flowers. LPS-stimulated macrophages treated with DE-R (100 μg/mL) and DE-F (200 μg/mL), drastically decreased the IL-6 production in 87.6 ± 0.9% and 83.2 ± 6.8%, reaching similar values obtained for dexamethasone. It is important to notice that DE-L (78 μg/mL), studied at lower concentrations, also reduced the IL-6 production in 81.1 ± 6.7%. EE-L (100 μg/mL), EE-R (200 μg/mL) and EE-F (200 μg/mL) demonstrated a similar activity to reduce the IL-6 production (77.8 ± 3.4%, 77.4 ± 3.4%, and 80.1 ± 5.3%, respectively). The same behavior was observed for AE-L (200 μg/mL, 51.2 ± 9.1%), AE-R (200 μg/mL, 54.6 ± 4.3%), and AE-F (250 μg/mL, 61.8 ± 12.3%). Analyzing all the *E. purpurea* extracts, the reduction in IL-6 production was more pronounced with DE-L, followed by DE-R, DE-F, EE-L ≈ EE-R, EE-F, AE-R ≈ AE-L, and AE-F.

Diclofenac (10 μM), celecoxib (10 μM), salicylic acid (10 μM), and dexamethasone (10 μM) presented a statistically significant capability to reduce the IL-1β production in 32.9 ± 2.8%, 36.3 ± 10.2%, 39.1 ± 3.8%,49.2 ± 5.9%, respectively (Figure 5B). *E. purpurea* extracts showed a capacity to reduce the IL-1β production by LPS-stimulated macrophages, although a concentration-dependent reduction was not observed in all extracts (Figure 5B). DCM and EtOH originated the strongest *E. purpurea* extracts for the reduction in IL-1β production. Extracts prepared with water presented the lowest bioactivity to reduce this pro-inflammatory cytokine (around 1.3 times lower). Flowers exhibited a higher capacity to decrease the IL-1β production, followed by roots and leaves, throughout the three solvents. AE-F (250 μg/mL), EE-F (100 μg/mL), EE-R (200 μg/mL), DE-F (100 μg/mL), and DE-R (50 μg/mL) showed a significant reduction in IL-1β production (54.9 ± 5.6%, 58.3 ± 3.5%, 63.3 ± 4.2%, 72.7 ± 15.0%, and 55.2 ± 1.7%, respectively), having a stronger anti-inflammatory activity than the anti-inflammatory drugs used as controls. EE-L (25 μg/mL) exhibited similar bioactivity in comparison with controls. With a similar or lower bioactivity than the clinical controls, AE-L (250 μg/mL), AE-R (250 μg/mL), EE-L (25 μg/mL), DE-L (39 μg/mL) reduced the IL-1β production, respectively, in 23.7 ± 4.5%, 26.4 ± 8.4%, 31.8 ± 2.0%, and 30.0 ± 14.6%. Analyzing all the *E. purpurea* extracts, the reduction in IL-1β production was more pronounced with DE-F, followed by EE-R, EE-F, DE-R ≈ AE-F, EE-L ≈ DE-L, and AE-R ≈ AE-L. Surprisingly, the levels of IL-1β were considerably enhanced in the presence of EE-L in the highest tested concentration (200 μg/mL, 157.4 ± 21.5%, data not presented in Figure 5B). An increase in IL-1β amount with extract concentration was also observed in the EE-F (200 μg/mL, 33.6 ± 1.5%) and DE-R (100 μg/mL, 62.7 ± 0.6%).

Dexamethasone (10 μM) was the most efficient control in the reduction in TNF-α production (48.1 ± 2.2%; Figure 5C). However, in this study, diclofenac (10 μM), salicylic acid (10 μM), and celecoxib (10 μM) did not show significant ability to reduce the TNF-α concentration in the culture medium, presenting a reduction in the TNF-α production of 15.9 ± 10.6%, 22.2 ± 5.1% and 19.9 ± 6.7%, respectively. Conversely, the majority of *E. purpurea* extracts were able to significantly reduce TNF-α production. Only EE-R and DE-L were not significantly capable of reducing this pro-inflammatory cytokine at any tested concentration. In this case, water produced more powerful *E. purpurea* extracts. Their bioactivity was approximately 1.3 times better than EtOH and DCM, which exhibited a comparable reduction in TNF-α. There is no tendency for the highest reduction in TNF-α production related to the organ of the plant. AE-F (250 μg/mL) was the most powerful extract in the reduction in TNF-α (55.3 ± 4.5%), being the activity of this extract comparable with dexamethasone. At the same concentration, AE-R and AE-L also demonstrated the ability to significantly reduce TNF-α amount in 35.3 ± 11.6% and 22.7 ± 4.0%, respectively. EE-L (100 μg/mL) and EE-F (100 μg/mL) had the ability to decrease the TNF-α production in 34.9 ± 12.0% and 29.2 ± 9.9%, respectively. DE-R (100 μg/mL) and DE-F (100 μg/mL) led to a reduction in TNF-α production in 28.8 ± 2.9% and 23.5 ± 4.0%, respectively. Analyzing all the *E. purpurea* extracts, the reduction in IL-1β production was more pronounced with AE-F, followed by AE-R ≈ EE-L, EE-F ≈ DE-R, DE-F ≈ AE-L, EE-R, and DE-L. The levels of TNF-α were considerably enhanced in the presence of DE-F in the highest tested concentration (200 μg/mL, 153.4 ± 20.3%, data not presented in Figure 5C). An increase in TNF-α amount with extracts concentration was also observed in EE-L (200 μg/mL, 102.1 ± 13.8%) and EE-F (200 μg/mL, 79.4 ± 7.7%).

### 2.5. Effect of E. purpurea Extracts on ROS/RNS Generation

#### 2.5.1. Non-Stimulated Macrophages

**Figure 6:**
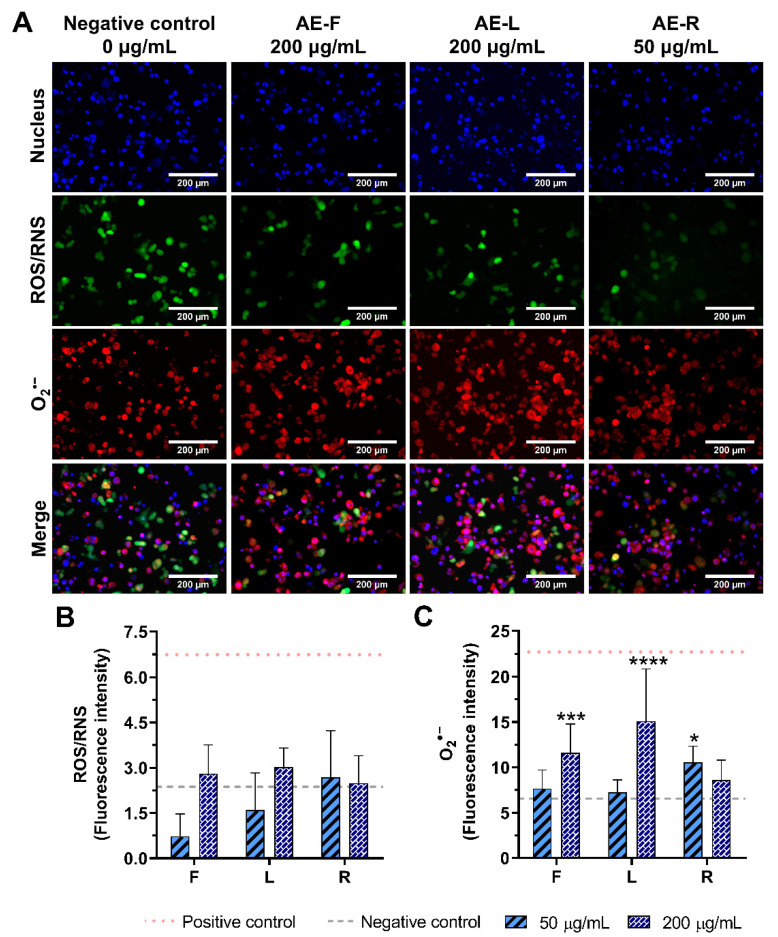
Intracellular ROS/RNS (green) and O_2_^•−^ (red) production in non-stimulated macrophages (nucleus in blue) in the absence (0 μg/mL) and in the presence of AE (200 μg/mL) obtained from *E. purpurea* flowers (F), leaves (L) and roots (R) cultured for 24 h (A). Fluorescence intensity of ROS/RNS (**B**) and O_2_^•−^ (**C**) was measured using ImageJ software. Non-stimulated macrophages produced a basal amount of ROS/RNS and O_2_^•−^ (grey dashed line, negative control) and LPS-stimulated macrophages produced a higher amount of ROS/RNS and O_2_^•−^ (red dotted line, positive control). Statistically significant differences are * (*p* < 0.0232), *** (*p* < 0.0010), and **** (*p* < 0.0001) in comparison with the negative control (non-stimulated macrophages without treatment) for each different tested extract.

As AE were the only extracts able to stimulate macrophages to produce pro-inflammatory cytokines, the intracellular levels of ROS/RNS and O_2_^•−^ production in the presence of these extracts were investigated (Figure 6 and Supplementary Figure S9). Non-stimulated macrophages (0 μg/mL, negative control) produced basal quantities of ROS and superoxide (Figure 6A). Intracellular levels of ROS/RNS were not significantly increased with the incubation of non-stimulated macrophages with AE at 50 or 200 μg/mL (Figure 6B). On the other hand, intracellular O_2_^•−^ levels were significantly increased in the presence of AE. AE-L (200 μg/mL) demonstrated a higher pro-inflammatory activity in the generation of O_2_^•−^, achieving the values obtained for the inflammatory state of LPS-stimulated macrophages (Figure 6C). AE-R at the lowest concentration (50 μg/mL) showed similar pro-inflammatory activity to AE-F at 200 μg/mL. Conversely, to AE-R, AE-F and AE-L presented a concentration-dependent pro-inflammatory activity.

#### 2.5.2. LPS-Stimulated Macrophages

**Figure 7:**
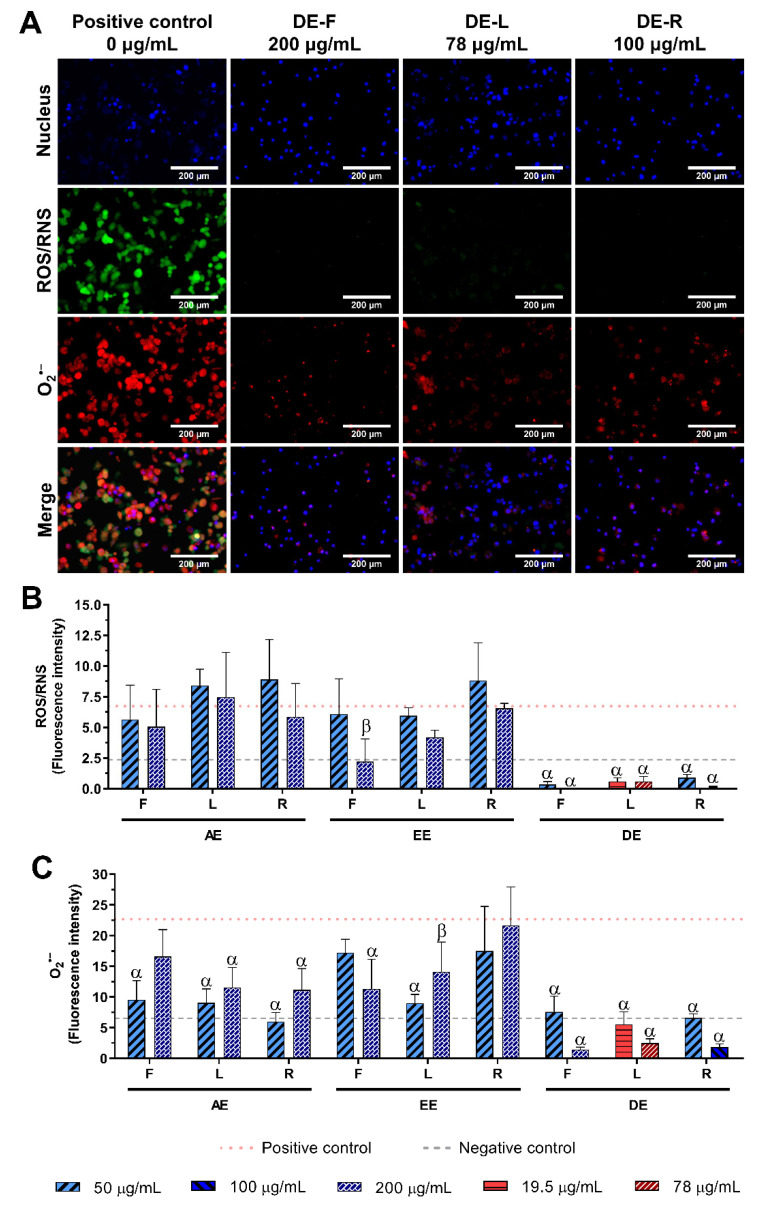
Intracellular ROS/RNS (green) and O_2_^•−^ (red) production in LPS-stimulated macrophages (nucleus in blue) in the absence (0 μg/mL) or in the presence of DE obtained from *E. purpurea* flowers (F), leaves (L) and roots (R) cultured for 24 h (**A**). Fluorescence intensity for ROS/RNS (**B**) and O_2_^•−^ (**C**) was measured using ImageJ software. Non-stimulated macrophages produced a small amount of ROS/RNS and O_2_^•−^ (grey dashed line, negative control) and LPS-stimulated macrophages produced a higher amount of ROS/RNS and O_2_^•−^ (red dotted line, positive control). Statistically significant differences are β (*p* < 0.0047), and α (*p* < 0.0001) in comparison to the positive control (LPS-stimulated control without treatment) for each different tested extract.

The reduction in intracellular levels of ROS/RNS and O_2_^•−^ in LPS-stimulated macrophages incubated with *E. purpurea* extracts at two different concentrations were evaluated (Figure 7 and Supplementary Figures S10–S12). As we previously mentioned, non-stimulated macrophages produced a basal amount of intracellular ROS and O_2_^•−^ (Figure 6), which was significantly increased by LPS-stimulation of the cells (Figure 7A).

The intracellular levels of ROS/RNS were only drastically reduced by DE at all tested concentrations, and the EE-F at 200 μg/mL, reaching similar or inferior levels to the non-stimulated macrophages (Figure 7B). All AE, EE-L, and EE-R did not have the capacity to decrease the ROS/RNS generation. Within the DE, flowers were more powerful in the ROS/RNS reduction, followed by roots and leaves. Analyzing all the *E. purpurea* extracts, DE-F demonstrated the most potent bioactivity, followed by DE-R, DE-L, EE-F, EE-L, AE-F ≈ AE-R, EE-R, and AE-L.

The intracellular levels of O_2_^•−^ were drastically decreased in the presence of all *E. purpurea* extracts, reaching similar or inferior levels to non-stimulated macrophages, except EE-R (Figure 7C). DCM produced stronger extracts to prevent the O_2_^•−^ generation, followed by water and EtOH. No tendency regarding the organ of the plant was noticed between the different solvents. DE-F presented a comparable reduction in O_2_^•−^ generation to DE-R, followed by DE-L, at the two tested concentrations. EE-L, at 50 μg/mL, showed to be more promising extracts for this bioactivity than EE-F (200 μg/mL). AE-R, AE-L, and AE-F were more efficient in the reduction in O_2_^•−^ generation at 50 μg/mL than at higher concentrations. Comparing all *E. purpurea* extracts, DE-F, DE-R, and DE-L were the most robust extracts, followed by AE-R, EE-L, AE-L, AE-F, EE-F, and EE-R.

## 3. Discussion

Plants produce a large amount of secondary metabolites with different bioactivities and therapeutic value in the clinic [49]. Particularly, *E. purpurea* is traditionally known due to its immunomodulatory properties. Its extracts have been prepared using mainly hydroethanolic solutions [24,28,29,50,51,52,53,54,55] or water [29,32,56]. Few studies reported the isolation of compounds where the starting solvent was methanol [57,58] or n-hexane [59,60]. Moreover, most of the studies used maceration to extract the bioactive compounds [24,29,51,55]. Soxhlet apparatus [50,59,60], stirring [32], and reflux [27,28] were also reported. As *E. purpurea* presents several compounds at different concentrations in the different organs of the plant [61], it was hypothesized that the organic solvent DCM could recover more hydrophobic compounds, which could also exhibit stronger bioactivity. Water, EtOH, and DCM were used as solvents to obtain extracts with different compositions for the immunomodulatory activity assays.

The major disadvantages of the classical extraction methods are (i) the huge time consumption, (ii) the need for large volumes of solvent, and (iii) the use of high temperatures [62]. The ASE is an excellent extraction technique that overcomes the previous issues, working as a classical soxhlet apparatus. The fast extraction time (12–30 min), the reduced solvent consumption (15–50 mL), the low sample amount (2–20 g), the controlled extraction temperature and pressure, and the high extraction yields, make it a new and innovative green extraction technique that ensures excellent reproducibility [62]. Moreover, as the solvent volume is significantly lower compared to the classical extraction techniques, the time, energy, and water consumption required to evaporate the solvent is considerably reduced. Hence, six *E. purpurea* extracts from the different organs of the plant—flowers, leaves, and roots—were prepared using ASE with EtOH or DCM as an extraction solvent. As water has low volatility, the AE obtained from flowers, leaves, or roots were prepared under stirring at RT. High temperatures were avoided, to prevent possible degradation of the bioactive compounds present in the extracts.

Water, being a polar solvent, had the highest extraction yield, and its extracts were mainly composed of phenols/acids (Figure 1 and Table 1). Some alkylamides were also present in the AE. EtOH also promoted high extraction yields, with rich content of phenols/acids and some alkylamides. DCM, being less polar than water and EtOH, recovered a higher amount of alkylamides and a low number of phenols/acids. In fact, phenols/acids are hydrophilic compounds, while alkylamides presented in this kind of extracts are described as lipophilic compounds [42,44,63]. Therefore, these results are in agreement with the literature. Phenols/acids are predominantly located in aerial parts (flowers and leaves) [61,64]. Alkylamides are concentrated in roots [48], but, in this study, we detected that a comparable number was found in roots and flowers of *E. purpurea*. Binns et al. also obtained a similar number of alkylamides in roots (14) and aerial parts (15) for young *E. purpurea* (≤1 year) [61]. Nevertheless, we are aware that different levels and/or types of these compounds should be observed for each different *E. purpurea* extract. Overall, this study shows the most extensive and comprehensive list of phenols/acids and alkylamides present in various *E. purpurea* extracts.

The extraction yield and the number of phenols/acids and alkylamides identified were significantly influenced by the polarity of the solvent, as well as the plant’s organ used. Therefore, the nine *E. purpurea* extracts presented different bioactive compounds (Table 1). Similar to the previous literature, the LC-HRMS results indicate that the phenols/acids, due to their high polarity, eluted first under reversed-phase conditions, while alkylamides, which are less polar, eluted later [42,44]. The MS/MS spectra of the ${\left[M-H\right]}^{-}$ precursor ions of phenols/acids exhibited two main peaks, one for the deprotonated molecular ion and another for a proton-bound dimer of this compound [42,44]. The four phenolic/acidic compounds described in *E. purpurea* preparations, including caftaric acid, chlorogenic acid, caffeic acid, and chicoric acid, were found in the nine *E. purpurea* extracts herein prepared [44,45,65]. None of the extracts presented echinacoside and cynarin, being consistent with results obtained by Binns et al. for young *E. purpurea* (≤1 year) [61]. Other acids and phenols/acids, such as malic acid, vanillic acid, protocatechuic acid, quinic acid, vanillin, benzoic acid, p-coumaric acid, rutin, and quercetin were identified in the different *E. purpurea* extracts, according to the literature [31,57,66,67,68,69,70]. The MS/MS spectra of the ${\left[M+H\right]}^{+}$ precursor ions of alkylamides showed that the major sites of fragmentation in alkylamides are the C−N bounds of the amide functional group. This generates fragments corresponding to the loss of the alkyl group attached to the nitrogen and the loss of the entire amine portion of the molecule [42,44]. Herein, based on MS/MS spectra, it is possible to distinguish between the two types of alkylamides—isobutylamides and 2-methylbutylamides—present in *E. purpurea* extracts [42]. In the case of isobutylamides, the fragments observed in the MS/MS spectrum correspond to a loss of the isobutyl group (−56 u), the isobutylamide group (−73 u), and the amide portion (−101 u) [42,44]. In the case of 2-methylbutylamides, the fragments corresponding to a loss of the 2-methylbutyl group (−70 u), the 2-methylbutyl amine (−87 u), and the amide portion (−115 u) are detected in the MS/MS spectrum [42,44]. The fragments obtained from the cleavage of the C−C bonds of the main carbon chain, composed of many sites of unsaturation, are also frequently recognized in the MS/MS spectrum of alkylamides [42,44]. Many of the alkylamides are isomeric, and, consequently, coelution of structurally similar alkylamides is common. Therefore, its identification could be a challenge since mass data does not indicate the stereochemistry or bond position. However, it has been established previously that the E isomer elutes first than the Z isomer [47]. For instance, undeca-2E,4Z-diene-8,10-diynoic acid isobutylamide eluted at 20.2 min, and undeca-2Z,4E-diene-8,10-diynoic acid isobutylamide appeared at 20.5 min. Another alkylamide isomer, dodeca-2E,4Z-diene-8,10-diynoic acid isobutylamide (*t_R_* = 20.7 min) and dodeca-2Z,4E-diene-8,10-diynoic acid isobutylamide (*t_R_* = 20.9 min) showed similar behavior (Supplementary Table S3). Nevertheless, the unequivocal identification of phenols/acids and alkylamides requires isolation of the individual compounds, followed by several purification steps, and elucidation of its structure by NMR analysis. The assignment of the configuration of the double bounds is not possible with the present techniques, and the reported stereochemistry of the identified alkylamides is, therefore, only tentative. The elution sequence of the alkylamides is predominantly influenced by the chain length and the number of double and triple bonds [48]. For example, undeca-2E,4Z-diene-8,10-diynoic acid isobutylamide elutes first (*t_R_* = 20.2 min, C_15_H_19_NO) than dodeca-2E,4Z-diene-8,10-diynoic acid isobutylamide (*t_R_* = 20.7 min, C_16_H_21_NO) (Supplementary Table S3). On the other hand, undeca-2E/Z-ene-8,10-diynoic acid isobutylamide (*t_R_* = 20.4 min, 1 double bound and 2 triple bounds) elutes before than dodeca-2E,4E,8Z,10E/Z-tetraenoic acid isobutylamide (*t_R_* = 21.5 min, 4 double bound), followed by dodeca-2E,4E-dienoic acid isobutylamide (*t_R_* = 21.9 min, 2 double bound) (Supplementary Table S3). Polyacetylenic alkylamides (undeca-2E,4Z-diene-8,10-diynoic acid isobutylamide, undeca-2Z,4E-diene-8,10-diynoic acid isobutylamide, undeca-2E-ene-8,10-diynoic acid isobutylamide, dodeca-2Z,4E-diene-8,10-diynoic acid isobutylamide, dodeca-2E,4Z-diene-8,10-diynoic acid isobutylamide, dodeca-2Z,4E-diene-8, 10-diynoic acid isobutylamide, dodeca-2E,4Z-diene-8,10-diynoic acid 2-methylbutylamide, dodeca-2E-ene-8,10-diynoic acid isobutylamide, dodeca-2E,4E,10E-triene-8-ynoic acid isobutylamide, trideca-2E,7Z-diene-10,12-diynoic acid isobutylamide, trideca-2E,7Z-diene-8,10-diynoic acid isobutylamide) eluted first, followed by the polyenic tetraenes and dienes alkylamides (dodeca-2E,4E,8Z,10E/Z-tetraenoic acid isobutylamide, dodeca-2E,4E,8Z-trienoic acid isobutylamide, and dodeca-2E,4E-dienoic acid isobutylamide), which is in agreement with previous data from the literature [43,44].

Macrophages were used to evaluate the immunomodulatory activity of the different extracts [71]. These cells have a crucial role in the inflammatory process and the defense against infectious pathogens, regulating the secretion of pro-inflammatory cytokines and the production of ROS/RNS. However, a prolonged overproduction of these inflammatory mediators is observed in a chronic inflammatory response, leading to compromised tissue functions [72,73]. In contrast, in immunodeficiency diseases, the activation of the immune system is required to eliminate infectious pathogens [74].

In the first approach of this study, it was evaluated the effect of the different *E. purpurea* extracts on the enhancement of the production of inflammatory cytokines and ROS/RNS by non-stimulated macrophages. In the second approach, it was investigated the potential of the *E. purpurea* extracts to reduce cytokines and ROS/RNS generation under an inflammatory scenario. As in the clinic, usually, an anti-inflammatory drug is only prescribed if patients have an inflammatory condition established, a pretreatment with the extracts was not performed. Indeed, the main goal of this study was not to evaluate their protective role in, e.g., avoiding an inflammatory cellular response, but to confirm that the developed extracts can be used as effective anti-inflammatory formulations. Additionally, plant extracts may affect the LPS/TLR4 signalling if added as a pretreatment [40,41]. Consequently, the results obtained from assays based on the pretreatment regimen cannot be directly related to the anti-inflammatory activity of the extracts. Therefore, this experimental design leads to more reliable and accurate results about the anti-inflammatory activity of the extracts. For both studies, cytocompatibility assays were performed to investigate the cytotoxicity of the *E. purpurea* extracts at different concentrations. Generally, the *E. purpurea* extracts were cytocompatible with non-stimulated (Figure 2) and LPS-stimulated macrophages (Figure 3). Moreover, a macrophage like-phenotype was observed when the cells were cultured with the *E. purpurea* extracts for both non-stimulated (Supplementary Figures S1–S4) and LPS-stimulated macrophages (Supplementary Figures S1,S5–S7). Indeed, only DE-R in the highest tested concentration significantly affected the macrophages’ metabolic activity, DNA concentration, protein synthesis, and morphology, demonstrating its cytotoxicity when present in high amounts.

AE showed the ability to induce the production of the pro-inflammatory cytokines IL-1β, IL-6, and TNF-α by non-stimulated macrophages (Figure 4). Furthermore, AE also promoted the intracellular generation of O_2_^•−^ (Figure 6 and Supplementary Figure S9), evidencing their immunostimulatory capacity of macrophages. AE-F was the most effective immunostimulatory extract. In fact, AE-F comprises more phenols/acids and alkylamides (7 phenols/acids and 14 alkylamides) than AE-R (3 phenols/acids and 16 alkylamides) or AE-L (only composed of phenols/acids and other acids). Therefore, it is possible to hypothesize that a synergistic effect between bioactive compounds is at the origin of this strong immunostimulatory activity, since lower immunostimulatory activity was observed in AE-L. This is also corroborated by the absence of immunostimulatory activity of DE-R, the richest alkylamide extract in this study (Supplementary Figure S9). Moreover, water promoted the recovery of other compounds besides the studied ones, such as polysaccharides, which can also have an influence in the immunostimulatory activity here demonstrated [75]. Consequently, these promising results suggest the application of AE, mainly AE-F, as a potential formulation to use in immunodeficiency disorders, where the stimulation of the immune system is insufficient.

Our results also indicate that all nine *E. purpurea* extracts drastically reduced IL-6 production by LPS-stimulated macrophages (Figure 5A). The DE extracts enriched in alkylamides as described by LC-HRMS were the most effective in the reduction in IL-6 production. Despite DE-R and DE-F containing a higher number of alkylamides, DE-L with only 8 alkylamides presented a stronger anti-inflammatory activity. Consequently, the set of alkylamides presented in this extract, including dodeca-2E,4Z-diene-8,10-diynoic acid isobutylamide, dodeca-2E,4E,8Z,10E/Z-tetraenoic acid isobutylamide, and dodeca-2E,4E-dienoic acid isobutylamide, with recognized anti-inflammatory activity [76], can be responsible for the highest effect of this extract. IL-1β production was also significantly reduced in the presence of all *E. purpurea* extracts (Figure 5B). In this case, DE-F and EE-R were the most potent extracts in decreasing IL-1β production. These extracts showed having similar composition, being EE-R enhanced with phenols/acids. Interestingly, DE-R, with a similar number of identified alkylamides and phenols/acids to the DE-F, showed 1.3 times lower activity. Thus, a specific alkylamide or phenol/acids in specific amounts should be directly related to the reduction in the IL-1β production. Finally, TNF-α production was efficiently inhibited by seven *E. purpurea* extracts (Figure 5C). AE-F and AE-R were the most effective, while DE showed an intermediate bioactivity. Therefore, a synergetic effect between alkylamides and phenols/acids may also be the reason for the reduction in TNF-α production.

There are several studies reporting the time-dependent gene expression and cytokine secretion after LPS stimulation (e.g., 0–30 h) on immune cells (e.g., monocytes and macrophages) [77,78,79,80]. Particularly, Chanput et al. reported that the exposure of THP-1 macrophages to LPS strongly induces IL-6, IL-1β, and TNF-α gene expression and protein secretion over time [77]. Moreover, they also demonstrate that the onset of up-regulation of cytokine genes is within 2 h of LPS-stimulation and the cytokine secretion is approximately 1 h after [77]. In addition, the relative order in abundance of cytokines is deeply correlated with the order of their responsive genes, being TNF-α production induced faster, followed by IL-1β and then IL-6 [77]. Indeed, although IL-6, IL-1β, and TNF-α are triggered by the same transcription factor [3], the kinetics of gene transcription and transduction does not occur at the same time, being the induction of TNF-α mRNA faster than the IL-6 mRNA [78]. Similar to in vitro studies, in a human experimental systemic inflammatory model, where a standard reference of *Escherichia coli* endotoxin was injected, TNF-α levels showed a peak in plasma within 90 min after LPS administration [79,80], whereas IL-6 peak appeared after 120 min [80]. In another study, IL-1β concentration peak was observed after TNF-α, but before IL-6 [81]. These observations are correlated with the pattern of cytokine levels found in here in this study after the addition of *E. purpurea* extracts. The LPS-stimulated macrophages produced and released TNF-α and IL-1β within 2 h. At the moment that the *E. purpurea* extracts were added (2 h after LPS addition), macrophages started the production of IL-6. Therefore, the inhibition of this cytokine was more pronounced than the others since its cascade was immediately inhibited. Indeed, as TNF-α and IL-1β inflammatory cascades initiated earlier, *E. purpurea* extracts will present a minor effect on their inhibition. Nevertheless, *E. purpurea* extracts were able to significantly decrease the production of these pro-inflammatory cytokines, reaching similar or lower amounts than the well-known tested NSAIDs (diclofenac, salicylic acid, and/or celecoxib) and the strong corticosteroid (dexamethasone). Thus, formulations of *E. purpurea* extracts can be a promising therapeutic strategy for the reduction in key cytokines in the inflammatory process. Unexpectedly, the secretion of IL-1β was significantly enhanced in the presence of EE-L (200 μg/mL). An equivalent behavior was observed for DE-F (200 μg/mL) for TNF-α. These results suggest that there is a specific amount of extract that could, in fact, exert its anti-inflammatory activity. Upon a threshold of concentration, the extracts are no longer effective since EE-L and DE-F did not promote cytokine production in non-stimulated macrophages (Supplementary Figure S8). Moreover, EE-F, EE-L, and DE-R showed an increase in IL-1β and TNF-α amounts with the increase in concentration, corroborating this hypothesis (Figure 5).

*E. purpurea* extracts showed to be strong and promising antioxidant formulations, able to protect DNA and cell membranes since the intracellular generation of ROS/RNS, and specifically O_2_^•−^, was suppressed under oxidative stress conditions (Figure 7 and Supplementary Figures S10–S12). Moreover, DE were able to strongly reduce both ROS/RNS and O_2_^•−^ generation in LPS-stimulated macrophages, reaching considerably inferior levels than non-stimulated macrophages. As previously mentioned, DE are alkylamide-enriched extracts, which may be in the origin of the observed bioactivity. Furthermore, all the *E. purpurea* extracts demonstrated a capacity to strongly reduce the intracellular O_2_^•−^ generation. This is a very promising result, because O_2_^•−^ can rapidly combine with NO to form RNS, such as peroxynitrite. The RNS, in turn, induces nitrosative stress, which accelerates the pro-inflammatory burden of ROS [82]. Therefore, the initial neutralization of the O_2_^•−^, will mitigate the ROS production, and, consequently, the protection of DNA, lipids, and other biomolecules can be observed.

As expected, DCM, with the lowest extraction yield, showed to be an excellent solvent to obtain potent extracts against the inflammatory process. Moreover, DE was enriched in alkylamides, which may be the main active principle of *E. purpurea* extracts in the anti-inflammatory activity. Taking all the results together, EE-F, DE-F, and DE-R demonstrated to be promising high-quality anti-inflammatory extracts.

## 4. Materials and Methods

### 4.1. Materials

Purple coneflower (*E. purpurea*) was purchased from Cantinho das Aromáticas (Vila Nova de Gaia, Portugal), in May 2017. The plants were immediately transferred to the soil and were let to grow following a sustainable agriculture procedure (41°37′04.5″ N, 7°16′14.4″ W). After one year of cultivation, the flowers and leaves were collected in a full bloom phase (June and July 2018), while the roots, including rhizomes, were harvested in the autumn (October 2018). The plants were dried in the dark and stored at RT protected from the light. A voucher specimen of roots (DB-15-EPR) and aerial parts (DB-16-EPT) was deposited at the Department of Biology, University of Minho, Portugal. EtOH and DCM were obtained from Fisher Scientific, Portugal. Ultra-pure water was obtained from a Milli-Q^®^ Direct Water Purification System (Milli-Q Direct 16, Millipore). Acetonitrile (ACN, HPLC grade), methanol (HPLC grade), formic acid (99%, analytical grade), phorbol 12-myristate 13-acetate (PMA), LPS (*Escherichia coli* O26:B6), and high-purity standards of echinacoside, chicoric acid, caftaric acid, caffeic acid, chlorogenic acid, and cynarin were obtained from Sigma-Aldrich, Portugal. Echinacea isobutylamide standards kit, composed of undeca-2E/Z-ene-8,10-diynoic acid isobutylamide, dodeca-2E-ene-8,10-diynoic acid isobutylamide, and dodeca-2E,4E-dienoic acid isobutylamide, was acquired from ChromaDex, Los Angeles, CA, USA, California. Highly-purity standard dodeca-2E,4E,8Z,10E/Z-tetraenoic acid isobutylamide was obtained from Biosynth Carbosynth. Roswell Park Memorial Institute (RPMI)-1640 medium, fetal bovine serum (FBS), antibiotic/antimycotic solution, Dulbecco’s phosphate-buffered saline (DPBS), formalin 10% (*v*/*v*), Quant-iT PicoGreen dsDNA Kit and Micro BCA protein assay kit were purchased from Thermo Fisher Scientific, Portugal. Dimethyl sulfoxide (DMSO) was obtained from VWR. AlamarBlue^®^ was purchased from Bio-Rad. Human IL-6, IL-1β, and TNF-α DuoSet Enzyme-linked immunosorbent assay (ELISA) kits and DuoSet ELISA Ancillary Reagent Kit 2 were purchased from R&D Systems, USA, Minneapolis. Cellular ROS/Superoxide detection assay kit was obtained from Abcam, USA, Boston. DAPI (4′,6-diamidino-2-phenylindole) was purchased from Biotium, Fremont, CA, USA. Coffee filter paper N4 was acquired in a local market.

### 4.2. Bioactive Compounds Extraction

Dried *E. purpurea* flowers (F), leaves (L), and roots (R) were ground using a blender (Picadora Clássica 123 A320R1, Moulinex, Lisbon, Portugal) before bioactive compounds extraction. EE and DE were obtained using an ASE 200 (Dionex Corp., Vigo, Spain). About 2–5 g of each sample was weighed and mixed with diatomaceous earth, a dispersant and drying agent. Then, they were loaded into stainless-steel extraction cells and held down to remove any residual free space. Cellulose filters were inserted into the bottom of those extraction cells before loading the sample to prevent the presence of suspended particles in the extract. All extractions were performed using two cycles, at constant pressure (1500 psi) for 30 min, at the minimum temperature allowed by the equipment (40 °C). The EE and DE solutions were collected into vials and then the organic solvent was evaporated using gas nitrogen.

AE were prepared by stirring 20 g of sample in 150 mL of ultra-pure water at RT for 24 h. The water was changed after 12 h of the extraction process. After extraction, AE was filtrated using a coffee filter paper N4. Both solutions were mixed, frozen at −80 °C and then freeze-dried (Lyoquest −85 °C Plus Eco, Telstar, Terrassa, Spain).

Once dried, the extraction yield for all the extracts was calculated based on the dry extract weight obtained compared to the initial mass of dry plant material used for extraction. The extraction yield of each *E. purpurea* extract is expressed in percentage (%). The extracts were stored at −80 °C until further assays.

### 4.3. Characterization of E. purpurea Extracts Composition

#### 4.3.1. Preparation of *E. purpurea* Extracts and Standards

A stock solution of 5 mg/mL of each *E. purpurea* extract was prepared. AE were dissolved in ultra-pure water, while EE and DE were prepared in methanol. The *E. purpurea* extracts solutions were centrifuged at 10,000× *g* for 5 min (ScanSpeed Mini, Labogene, Lynge, Denmark) and the supernatant was collected.

A stock solution of 1 mg/mL of all standards was prepared and stored in amber bottles at −80 °C. All the standards were prepared in methanol, except caffeic acid, which was prepared in ethanol. A mixture solution of all standards was prepared at a final concentration of 5 μg/mL each.

#### 4.3.2. LC-HRMS Analysis

The LC−HRMS analysis was performed on UltiMate 3000 Dionex ultra-high-performance liquid chromatography (UHPLC, Thermo Scientific, Lisbon, Portugal), coupled to an ultrahigh-resolution quadrupole—quadrupole time-of-flight (UHR–QqTOF) mass spectrometer (Impact II, Bruker). The chromatographic separation was performed on an Acclaim RSLC 120 C18 analytical column (100 mm x 2.1 mm i.d.; 2.2 µm, Dionex, Lisbon, Portugal). The mobile phase was composed by (A) water containing 0.1% formic acid and (B) ACN containing 0.1% formic acid. The gradient program was as follows: 0 min, 95% A; 10 min, 79% A; 14 min, 73% A; 18.3 min, 42% A; 20 min, 0% A; 24 min, 0% A; 26 min, 96% A. The flow rate was of 0.25 mL/min, and the column was kept at 35 °C. The injection volume was 2 μL. The MS analysis of the phenolic/acidic compounds was set using electrospray ionization (ESI) in negative ionization mode due to their acidic character [42]. As the alkylamides are slightly basic, they were detected in the positive ion mode [42]. Spectra was acquired over a range from m/z 20 to 1000 in an Auto MS scan mode. The selected parameters were as follows: capillary voltage, 2500 V (negative mode, phenols/acids) and 4500 V (positive mode, alkylamides); drying gas temperature, 200 °C; drying gas flow, 8.0 L/min; nebulizing gas pressure, 2 bar; collision cell energy, 5.0 eV; collision radio frequency (RF), 300 Vpp; transfer time, 70 μs; and prepulse storage, 5 μs. Post-acquisition internal mass calibration used sodium formate clusters, being sodium formate delivered by a syringe pump at the start of each chromatographic analysis. The LC-HRMS acquired data were processed using Bruker Compass DataAnalysis 5.1 software (Bruker) to extract the mass spectral features from the sample raw data. Standards were commercially available for echinacoside, chicoric acid, caftaric acid, caffeic acid, chlorogenic acid, cynarin, undeca-2E/Z-ene-8,10-diynoic acid isobutylamide, dodeca-2E-ene-8,10-diynoic acid isobutylamide, dodeca-2E,4E-dienoic acid isobutylamide, and dodeca-2E,4E,8Z,10E/Z-tetraenoic acid isobutylamide. Therefore, the identification of these compounds in the *E. purpurea* extracts was confirmed by their retention times (*t*_R_, min), mass-to-charge ratio (*m*/*z*) of the molecular ion, and MS/MS fragmentation patterns. Supplementary Table S1 summarizes the mass spectra information for all the standards obtained by LC-HRMS. For phenols/acids and alkylamides for which standards were not available, the potential candidates to a specific molecule were assigned by comparing the theoretical and published MS/MS fragments pattern with the obtained MS/MS spectra pattern, and by analyzing the elution order of alkylamides present in the literature [42,43,44,45,46,47,48].

### 4.4. E. purpurea Extract Solutions

AE was dissolved in complete RPMI 1640 medium (cRPMI, RPMI medium supplemented with 10% FBS and 1% antibiotic/antimycotic solution), and EE and DE were dissolved in DMSO. Due to the different extract solubility, the stock solutions, sterilized with a 0.22 µm filter, were 12.8 mg/mL for AE (F, L, and R), 60.0 mg/mL for EE (F, L, and R), DE-F, and DE-R and 23.5 mg/mL for DE-L. Then, serial dilutions were made with RPMI. The final concentrations tested were of 25.0, 50.0, 100.0, 200.0 and 250.0 μg/mL for AE (F, L and R); 12.5, 25.0, 50.0, 100.0 and 200.0 μg/mL for EE (F, L and R) and DE-F and DE-R; and 4.9, 9.8, 19.5, 39.0 and 78.0 μg/mL for DE-L. The percentage of DMSO in the well for the maximum concentration of extracts was 0% for AE, 0.33% for EE (F, L, and R), DE-L and DE-F, and 0.53% for DE-R.

### 4.5. Pro-Inflammatory Activity Evaluation

The pro-inflammatory activity of the *E. purpurea* extracts was evaluated using a human peripheral blood monocyte cell line (THP-1), obtained from American Type Culture Collection (ATCC^®^ TIB-202™), according to the procedure described by Vieira et al. [83]. Briefly, THP-1 cell line, at passages 10–13, was cultured in cRPMI, at 37 °C in a humidified atmosphere of 5% CO_2_. THP-1 cell line was seeded at a density of 1 × 10^6^ cells/mL in adherent 24-wells culture plates. For the induction of THP-1 cell differentiation, RPMI medium containing 100 nM PMA was added and incubated for 24 h [84]. After this period, the medium containing non-attached cells was removed by aspiration, and the adherent cells were washed twice with warm cRPMI medium. To ensure the reversion of monocyte to a resting macrophage phenotype, the cells were incubated for an additional period of 48 h in cRPMI without PMA. Afterward, the medium was changed and each *E. purpurea* extract at different concentrations (see Section 4.4) were added to the non-stimulated macrophages. After 24 h, the culture medium was harvested (the triplicates were mixed and homogenized) and stored aliquoted at −80 °C until cytokines quantification. The cells were washed with warm sterile DPBS and the metabolic activity, DNA quantification and total protein content were determined as described below (see Section 4.7). Cell morphology was analyzed before collecting medium under an inverted microscope (AxioVert A1 FL LED, Zeiss, Göttingen, Germany). Controls containing the same percentage of DMSO in the maximal concentration of extracts were also tested and did not affect the cell viability.

### 4.6. Anti-Inflammatory Activity Evaluation

THP-1 cells were seeded and cultured as previously described (see Section 4.5). After the total reversion of monocyte to macrophage phenotype, macrophages were stimulated with 100 ng/mL of LPS in a fresh medium. After 2 h, each *E. purpurea* extract at different concentrations (see Section 4.4) was added to the LPS-stimulated macrophages and incubated for 22 h. Afterward, the culture medium was harvested and stored, as previously described. Then, the cells were washed with warm sterile DPBS and the cell morphology, metabolic activity, DNA quantification, total protein content, and cytokine quantification were determined, as described below (see Section 4.7). LPS-stimulated macrophages cultured without extracts (no treatment, 0 μg/mL) were used as a positive control of cytokine production. Dexamethasone (10 µM), diclofenac (10 µM), salicylic acid (10 µM), and celecoxib (10 µM), dissolved in ethanol, were used as positive controls for inhibition of cytokine production. Negative controls of cells without LPS (no stimulation) were also tested. Controls containing the same percentage of DMSO (see Section 4.5) in the maximal concentration of extracts were also tested and showed not to affect the cell viability.

### 4.7. Metabolic Activity, DNA Quantification, and Total Protein Content

The metabolic activity, DNA concentration, and total protein content of non-stimulated and LPS-stimulated macrophages incubated with *E. purpurea* extracts were determined using the alamarBlue assay, fluorimetric dsDNA quantification kit, and Micro BCA protein assay kit, as previously described by us [84,85,86]. The results of metabolic activity are expressed in percentage related to the control. DNA and total protein contents are expressed in relative concentrations to the control.

### 4.8. Cytokine Quantification

The amountof different cytokines produced by macrophages, namely IL-1β, IL-6, and TNF-α, in the culture medium was assessed using different ELISA kits, according to the manufacturer’s instructions. The values obtained were normalized by the respective DNA concentration. The results obtained for the determination of the anti-inflammatory activity are expressed in percentage related to the control [87].

### 4.9. Cellular ROS/RNS/O_2_^•−^ Detection Assay

Oxidative stress in the presence or absence of *E. purpurea* extracts was investigated using Cellular ROS/Superoxide detection assay kit. Briefly, the THP-1 cell line was seeded (1 × 10^5^ cells/mL) in an adherent 24-wells culture as previously described for pro- and anti-inflammatory assays (see Section 4.5 and Section 4.6). After incubation with *E. purpurea* extracts (50 and 200 μg/mL for AE-F, L, and R-, EE -F, L, and R- and DE-F; 50 and 100 μg/mL for DE-R; and 19.5 and 78 μg/mL for DE-L), the supernatant was removed, and the cells were labeled with oxidative stress detection reagent (green, Ex/Em 490/525 nm) for detection of total ROS/RNS and O_2_^•−^ detection reagent (orange, Ex/Em 550/620 nm) for 1 h, at 37 °C in the dark. These nonfluorescent detection reagents diffuse into cells, where they can be oxidized by ROS/RNS and O_2_^•−^, converting to fluorescent probes. Then, the cells were fixed with 10% of formalin for 10 min and DAPI in a ratio of 1:1000 in DPBS was added for more 10 min. Between each step, the cells were carefully washed twice with 300 μL of DPBS. The fluorescent samples were analyzed using a Fluorescence Inverted Microscope with Incubation (Axio Observer, Zeiss, Göttingen, Germany). The fluorescence intensity was analyzed using ImageJ software. Changes in the fluorescence intensity relative to the control with or without LPS (0 μg/mL) were related to an increase or decrease in the generation of intracellular ROS/RNS and/or O_2_^•−^.

### 4.10. Statistical Analysis

Results are expressed as mean ± standard deviation (SD) of 3 independent experiments, with a minimum of 3 replicates for each condition. Statistical analyses were performed using GraphPad Prism 8.0.1 software. Analysis of variance (ANOVA) and Tukey’s multiple comparisons test were used for extraction yield. ANOVA and Dunnett’s multiple comparison method were used for cell assays. Differences between experimental groups were considered significant with a confidence interval of 99% whenever *p* < 0.01.

## 5. Conclusions

In this work, we demonstrated that *E. purpurea* extracts can modulate macrophage behavior. AE presented a dual activity, being capable of a pro- and anti-inflammatory/oxidant extract. The synergistic effect between bioactive compounds was proposed for immunostimulatory activity. AE-F, composed of phenols/acids and alkylamides, presented the highest bioactivity than AE-L, only containing phenols/acids, which suggests that different interactions between the compounds are responsible for the immunostimulatory activity. In addition, DE, alkylamide-enriched extracts, drastically reduced the main pro-inflammatory cytokines and ROS/RNS production, allowing for the suppression of the inflammatory response. Moreover, the *E. purpurea* extracts showed generally more robust anti-inflammatory activity than the conventional NSAIDs and corticosteroid used in the clinic. Therefore, *E. purpurea* extracts can be used to isolate new drugs to treat diseases related to an overproduction of inflammatory mediators, such as auto-immune diseases, as well as diseases where a boost of the immune system and inflammatory response is required, such as immunodeficiency diseases and cancer. Further fractionation of *E. purpurea* extracts is required to specifically determine which class of compounds present in the extracts may really exert the pro- and anti-inflammatory activity, as well as to prove the synergistic effect proposed here. Additionally, the determination of the levels of specific compounds should be calculated for the *E. purpurea* extracts that exhibited the highest bioactivity.

## Figures and Tables

**Table 1:** Overview of the identified compounds (phenols/acids and alkylamides) in *E. purpurea* extracts by LC-HRMS. F: flowers; L: leaves; R: roots; AE: aqueous extracts; EE: ethanolic extracts; DE: dichloromethanolic extracts.

Compound	AE			EE			DE		
	F	L	R	F	L	R	F	L	R
**Phenols/acids**									
Malic Acid	X	X	X	X	X	X	-	X	X
Vanillic acid	X	X	-	X	-	-	-	-	-
Protocatechuic acid	X	X	-	X	X	-	-	-	-
Caftaric acid	X	X	-	X	X	X	-	X	-
Chlorogenic acid	-	-	-	X	-	X	-	-	-
Quinic acid	-	-	-	-	-	X	-	-	-
Vanillin	-	-	-	-	X	-	-	-	-
Caffeic acid	-	-	-	X	X	X	X	X	X
Benzoic acid	X	X	X	X	X	X	X	X	X
Cynarin	-	-	-	-	-	-	-	-	-
Echinacoside	-	-	-	-	-	-	-	-	-
*p*-coumaric acid	X	X	-	X	-	X	-	-	X
Chicoric acid	X	X	X	X	X	X	X	X	-
Rutin	-	-	-	X	-	-	-	-	-
Quercetin	-	-	-	X	-	-	-	-	-
**Alkylamides**									
Dodeca-2E,4Z,10E-triene-8-ynoic acid isobutylamide	X	-	X	X	-	X	X	-	X
Dodeca-2E,4Z,10Z-triene-8-ynoic acid isobutylamide	X	-	X	X	-	X	X	-	X
Dodeca-2,4,10-triene-8-ynoic acid isobutylamide (isomer 1)	-	-	-	-	-	-	X	-	X
Dodeca-2E,4E,10Z-triene-8-ynoic acid isobutylamide	-	-	X	X	-	X	X	-	X
Dodeca-2Z,4E,10Z-triene-8-ynoic acid isobutylamide	-	-	X	-	-	X	-	-	X
Dodeca-2E,4E,10E-triene-8-ynoic acid isobutylamide	X	-	-	X	X	X	X	X	X
Undeca-2E,4Z-diene-8,10-diynoic acid isobutylamide	X	-	X	X	X	X	X	X	X
Undeca-2E/Z-ene-8,10-diynoic acid isobutylamide	X	-	-	X	-	-	X	-	-
Undeca-2Z,4E-diene-8,10-diynoic acid isobutylamide	-	-	X	-	-	X	-	-	X
Undeca-2E/Z,4Z/E-diene-8,10-diynoic acid 2-methylbutylamide	-	-	-	-	-	-	-	-	-
Pentadeca-2E,9Z-diene-12,14-diynoic acid 2-hydroxyisobutylamide	-	-	-	X	X	-	X	X	-
Dodeca-2E,4Z-diene-8,10-diynoic acid isobutylamide	X	-	X	X	-	X	X	X	X
Undeca-2E,4E-diene-8,10-diynoic acid isobutylamide	-	-	-	-	-	X	-	-	X
Dodeca-2Z,4E-diene-8,10-diynoic acid isobutylamide	-	-	X	-	-	X	-	-	-
Dodeca-2E-ene-8,10-diynoic acid isobutylamide	X	-	-	X	X	X	X	X	X
Trideca-2E,7Z-diene-10,12-diynoic acid isobutylamide	X	-	X	X	-	X	X	-	X
Dodeca-2,4-diene-8,10-diynoic acid 2-methylbutylamide	-	-	X	X	-	X	X	-	X
*Dodeca-2Z,4Z,10Z-triene-8-ynoic acid isobutylamide ^1^*	-	-	X	-	-	X	-	-	X
Trideca-2E,7Z-diene-10,12-diynoic acid 2-methylbutylamide	X	-	-	X	-	X	X	-	X
Dodeca-2E,4E,8Z,10E/Z-tetraenoic acid isobutylamide	X	-	X	X	X	X	X	X	X
Dodeca-2E,4Z,10E-triene-8-ynoic acid 2-methylbutylamide *OR* Dodeca-2E-ene-8,10-diynoic acid 2-methylbutylamide	X	-	X	X	-	X	X	-	X
Dodeca-2E,4E,8Z-trienoic acid isobutylamide (isomer 1)	-	-	-	X	-	-	X	-	-
Dodeca-2E,4E-dienoic acid isobutylamide (isomer 1)	-	-	-	-	X	-	-	-	-
Pentadeca-2E,9Z-diene-12,14-diynoic acid isobutylamide	-	-	X	X	X	X	X	X	X
Dodeca-2E,4E,8Z-trienoic acid isobutylamide	X	-	X	X	-	X	X	-	X
Trideca-2Z,7Z-diene-10,12-diynoic acid 2-methylbutylamide	-	-	-	-	-	X	-	-	X
Dodeca-2E,4E,8Z,10E/Z-tetraenoic acid 2-methylbutylamide	X	-	-	X	-	X	X	-	X
Hexadeca-2E,9Z-diene-12,14-diynoic acid isobutylamide	-	-	-	-	-	X	-	-	X
Dodeca-2E,4E,8Z-trienoic acid isobutylamide (isomer 2)	-	-	-	-	-	-	-	-	X
Dodeca-2E,4E-dienoic acid isobutylamide	X	-	X	X	X	X	X	X	X

^1^ This compound was not found in the literature. E/Z stereochemistry is indicated here in accordance with the existing literature [42,43,44,45,46,47,48], but it should be highlighted that without NMR spectra, it is not possible to conclusively distinguish between E and Z isomers. Smooth grey shaded corresponds to studied standards.

## Data Availability

Not applicable.

## Supplementary Materials

The following supporting information can be downloaded at: https://www.mdpi.com/article/10.3390/ijms232113616/s1.
