# Supplementary material for: On the Bioactivity of Echinacea purpurea Extracts to Modulate the Production of Inflammatory Mediators
Source: Int J Mol Sci. 2022 Nov 6;23(21):13616. doi: 10.3390/ijms232113616 (PMC9659013; doi:10.3390/ijms232113616)
Supplement: Supplementary file 1 [file ijms-23-13616-s001.zip › ijms-1941232-supplementary.pdf]

International Journal of *Molecular Sciences*

MDPI

Supplementary Material

On the Bioactivity of *Echinacea purpurea* Extracts to Modulate the Production of Inflammatory Mediators

Sara F. Vieira [1,2], Virgínia M. F. Gonçalves [3,4], Carmen P. Llaguno [5], Felipe Macías [5], Maria Elizabeth Tiritan [3,6,7], Rui L. Reis [1,2], Helena Ferreira [1,2,*] and Nuno M. Neves [1,2,*]

[1] 3B's Research Group, I3Bs—Research Institute on Biomaterials, Biodegradables and Biomimetics, University of Minho, Headquarters of the European Institute of Excellence on Tissue Engineering and Regenerative Medicine, AvePark, Parque de Ciência e Tecnologia, Zona Industrial da Gandra, Barco, 4805-017 Guimarães, Portugal
[2] ICVS/3B's—PT Government Associate Laboratory, 4710-057 Braga/Guimarães, Portugal
[3] TOXRUN—Toxicology Research Unit, University Institute of Health Sciences (IUCS), CESPU, CRL, 4585-116 Gandra, Portugal
[4] UNIPRO—Oral Pathology and Rehabilitation Research Unit, University Institute of Health Sciences (IUCS), CESPU, CRL, 4585-116 Gandra, Portugal
[5] Departamento de Edafoloxía e Química Agrícola, Facultade de Bioloxía, Universidade de Santiago de Compostela, 15782 Santiago de Compostela, Spain
[6] Interdisciplinary Centre of Marine and Environmental Research (CIIMAR), University of Porto, Terminal de Cruzeiros do Porto de Leixões, Avenida General Norton de Matos, S/N, 4450-208 Matosinhos, Portugal
[7] Laboratório de Química Orgânica e Farmacêutica, Departamento de Ciências Químicas, Faculdade de Farmácia da Universidade do Porto, Rua Jorge de Viterbo Ferreira 228, 4050-313 Porto, Portugal
* Correspondence: helenaferreira@i3bs.uminho.pt (H.F.); nuno@i3bs.uminho.pt (N.M.N.); Tel.: +351-253-510-913 (H.F.); +351-253-510-905 (N.M.N.); Fax: +351-253-510-909 (H.F. & N.M.N.)

Citation: Vieira, S.F.; Gonçalves, V.M.F.; Llaguno, C.P.; Macías, F.; Tiritan, M.E.; Reis, R.L.; Ferreira, H.; Neves, N.M. On the Bioactivity of *Echinacea purpurea* Extracts to Modulate the Production of Inflammatory Mediators. *Int. J. Mol. Sci.* **2022**, *23*, 13616. https://doi.org/10.3390/ijms232113616

Academic Editor: Massimiliano Gasparrini

Received: 13 September 2022
Accepted: 4 November 2022
Published: 6 November 2022

Publisher's Note: MDPI stays neutral with regard to jurisdictional claims in published maps and institutional affiliations.

Copyright: © 2022 by the authors. Licensee MDPI, Basel, Switzerland. This article is an open access article distributed under the terms and conditions of the Creative Commons Attribution (CC BY) license (https://creativecommons.org/licenses/by/4.0/).

Table S1. Characterization of caftaric acid, chlorogenic acid, caffeic acid, cynarin, echinacoside, chicoric acid, undeca-2E/Z-ene-8,10-diynoic acid isobutylamide, dodeca-2E-ene-8,10-diynoic acid isobutylamide, dodeca-2E,4E-dienoic acid isobutylamide, and dodeca-2E,4E,8Z,10E/Z-tetraenoic acid isobutylamide by LC-HRMS.

Standard compound	Chemical structure	Formula	Exact mass	Theoretical precursor ion (m/z)	Precursor ion (m/z)	t_R (min)	Product ions (m/z)
				**Phenols/acids**			
Caftaric acid	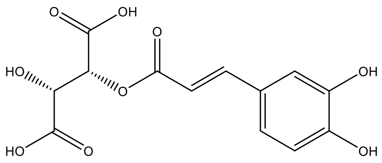	$C_{13}H_{12}O_9$	312.04813196	311.0403	311.0417 [M-H]-	4.8	179.0361 (100), 149.0100 (63), 135.0458 (35)
Chlorogenic acid	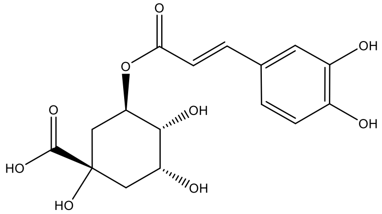	$C_{16}H_{18}O_9$	354.09508215	353.0873	353.0880 [M-H]-	6.4	191.0565 (100), 192.0599 (11), 353.0859 (5), 161.0240 (2), 179.0348 (1)
Caffeic acid	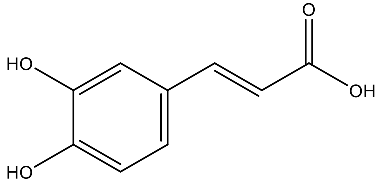	$C_9H_8O_4$	180.04225873	179.0345	179.0352 [M-H]-	7.3	135.0453 (100), 179.0354 (25), 136.0487 (13), 134.0372(9), 180.0384 (5), 107.0510 (2)
Cynarin	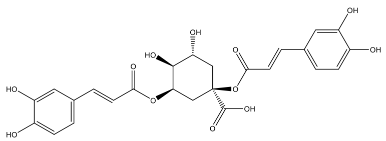	$C_{25}H_{24}O_{12}$	516.12677620	515.1190	515.1181 [M-H]-	8.6	353.0871 (100), 191.0561 (54), 179.0350 (53), 515.1170 (41), 354.0903 (20), 335.0753 (17), 516.1212 (10), 135.0452 (5), 161.0252 (5), 192.0598 (5), 173.0441 (5)

Echinacoside	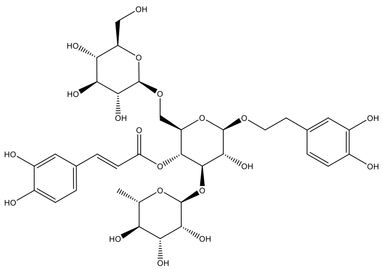	$C_{35}H_{46}O_{20}$	786.25824385	785.2504	785.2493 [M-H]-	9.3	785.2508 (100), 786.2537 (38), 161.0246 (22), 623.2174 (22), 787.2566 (11), 624.2247 (8), 132.0218 (1), 135.0458 (1)
Chicoric acid	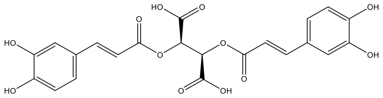	$C_{22}H_{18}O_{12}$	474.07982601	473.0720	473.0717 [M-H]-	10.6	149.0091 (100), 179.0343 (84), 311.0396 (64), 293.0295 (22), 135.0448 (8), 112.9864 (6)
**Alkylamides**							
Undeca-2E/Z-ene-8,10-diynoic acid isobutylamide	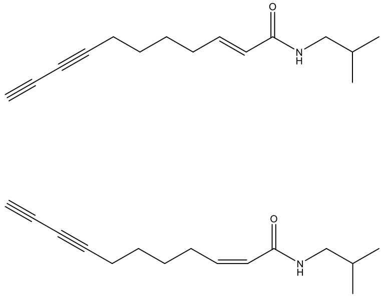	$C_{15}H_{21}NO$	231.16231	232.1701	232.1698 [M+H]+	20.4	57.0696 (100), 91.0541 (84), 79.0538 (46), 41.0382 (45), 65.0383 (32), 105.0698 (31), 115.0537 (23), 176.1068 (3)
Dodeca-2E-ene-8,10-diynoic acid isobutylamide	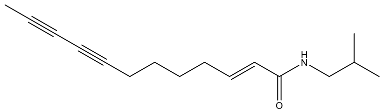	$C_{16}H_{23}NO$	245.17796	246.1858	246.1855 [M+H]+	20.8	57.0696 (100), 79.0536 (57), 91.0541 (39), 41.0381 (31), 105.0698 (28), 67.0537 (26), 77.038 (21), 81.0693 (20)
Dodeca-2E,4E,8Z,10E/Z-tetraenoic acid isobutylamide	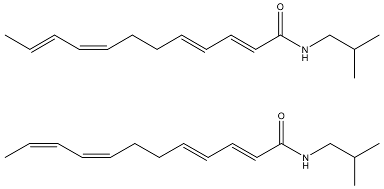	$C_{16}H_{25}NO$	247.19361	248.2014	248.2014 [M+H]+	21.2	57.0695 (100), 67.054 (44), 68.062 (34), 81.0692 (12), 41.0382 (12), 152.1065 (11), 79.0541 (9), 66.0463 (9), 167.1297 (4), 100.0763 (3)

Dodeca-2E,4E-dienoic acid isobutylamide	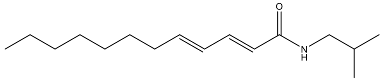	$C_{16}H_{29}NO$	251.22491	252.2327	252.2317 [M+H]+	21.9	57.0695 (100), 55.0538 (77), 81.0328 (52), 67.0539 (47), 69.0697 (45), 81.0691 (37), 95.0851 (22), 196.1692 (10)

Table S2. Phenolic/acid compounds tentatively identified in *E. purpurea* extracts by LC-HRMS (negative mode). F: flowers; L: leaves; R: roots; AE: aqueous extracts; EE: ethanolic extracts; DE: dichloromethanolic extracts.

**Proposed phenolic/acid compound**	**Precursor ion [M – H]⁻ (m/z)**	***t*R (min)**	**Product ions (m/z) [1]**
			**Aqueous Extracts obtained from Flowers**
Malic acid	133.0154	1.4	115.0040 (100), 71.0141 (72), 133.0149 (69), 72.9931 (27), 89.0241 (19), 43.0195 (7)
Vanillic acid	167.0357	3.6	152.0131 (100), 167.0350 (84), 123.0467 (36), 108.0241 (28)
Protocatechuic acid	153.0197	4.2	109.0298 (100), 153.0207 (26), 110.0322 (9), 81.0346 (7), 154.0885 (5), 108.0225 (3)
Caftaric acid	311.0407	4.8	179.0363 (100), 149.0102 (53), 135.0463 (27)
p-coumaric acid derivative	163.0410	6.4	119.0504 (100), 163.0401 (14), 120.0551 (8), 107.0134 (3), 146.0282 (2), 121.0298 (2)
Benzoic acid	121.0297	7.7	121.0296 (100), 122.0331 (14), 92.0258 (2), 120.0225 (2), 93.0349 (1)
p-coumaric acid	163.0409	9.6	119.0507 (100), 163.0400 (14), 120.0538 (7), 164.0437 (2), 164.0729 (2), 117.0342 (1), 93.0354 (1)
Chicoric acid	473.0729	10.6	149.0106 (100), 179.0357 (89), 311.0419 (46), 293.0321 (23), 135.0454 (10)
			**Aqueous Extracts obtained from Leaves**
Malic acid	133.0145	1.4	115.0038 (100), 71.0140 (69), 133.0143 (50), 72.9931 (25), 89.0241 (17)
Vanillic acid	167.0355	3.6	167.0361 (100), 123.0458 (78), 108.0214 (9), 152.0133 (4)
Protocatechuic acid	153.0203	4.2	109.0298 (100), 153.0192 (19), 108.0226 (19), 96.9610 (9), 110.0317 (9), 152.0298 (5), 123.0454 (5), 81.0346 (4)
Caftaric acid	311.0414	4.9	179.0361 (100), 149.0100 (63), 135.0457 (47)
p-coumaric acid derivative	163.0407	6.4	119.0505 (100), 163.0407 (17), 120.0532 (9), 119.45 (2), 93.0360 (2)
Benzoic acid	121.0294	7.7	121.0297 (100), 122.0343 (22), 81.0363 (4), 108.0233 (2), 120.5480 (2)
p-coumaric acid	163.0407	9.6	119.0505 (100), 163.0410 (14), 120.0537 (12), 147.0462 (3), 164.0444 (3)
Chicoric acid	473.0731	10.6	149.0101 (100), 179.036 (85), 311.042 (68), 293.0307 (32), 135.0461 (12), 112.9881 (12)
			**Aqueous Extracts obtained from Roots**

Malic acid	133.0149	1.4	115.0036 (100), 71.0139 (73), 133.0146 (40), 72.9930 (25), 89.0244 (17)
p-coumaric acid derivative	163.0410	6.4	119.0504 (100), 120.0523 (13), 163.0436 (9)
Benzoic acid	121.0292	7.8	121.0306 (100), 108.0224 (16), 122.0352 (10), 93.0341 (7)
Chicoric acid	473.0725	10.6	149.0094 (100), 311.0415 (84), 179.0357 (80), 293.0312 (26), 135.0453 (15), 112.9883 (10)
			**Ethanolic Extracts obtained from Flowers**
Malic acid	133.0143	1.3	115.0039 (100), 71.0139 (85), 133.0149 (53), 72.9929 (28), 43.0188 (11), 89.0238 (10)
Vanillic acid	167.0350	3.6	167.0355 (100), 123.0444 (60), 108.0203 (15), 152.0124 (9)
Protocatechuic acid	153.0199	4.2	109.0297 (100), 153.0202 (44), 108.0219 (12), 110.0333 (11), 154.0229 (3)
Caftaric acid	311.0409	4.8	179.0354 (100), 149.0094 (53), 135.0452 (40)
Chlorogenic acid	353.0868	6.4	191.0564 (100), 192.0595 (9), 161.0243 (3), 193.0612 (2), 353.0861 (1), 179.0335 (1)
Caffeic acid	179.0352	7.3	135.0453 (100), 179.0353 (28), 136.0485 (12), 134.0374 (9), 180.0394 (5), 107.0503 (1)
Benzoic acid	121.0289	7.7	121.0290 (100), 122.0318 (9), 120.0202 (2), 92.0245 (1)
p-coumaric acid	163.0404	9.6	119.0504 (100), 163.0407 (17), 120.0544 (14), 162.8385 (7), 117.0362 (3)
Chicoric acid	473.0731	10.6	149.0092 (100), 179.0352 (71), 311.0402 (43), 293.0292 (21), 112.9875 (13), 135.0452 (11)
Rutin	609.1451	10.8	609.1450 (100), 610.1469 (34), 300.0273 (25), 301.0343 (17), 611.1503 (7)
Rutin derivative	609.1446	11.0	609.1445 (100), 610.1480 (33), 300.0272 (23), 301.0345 (20), 611.1494 (8)
Quercetin	301.0349	16.8	301.0340 (100), 151.0036 (36), 178.9980 (28), 302.0386 (16), 121.0291 (7)
			**Ethanolic Extracts obtained from Leaves**
Malic acid	133.0143	1.4	115.0044 (100), 71.0138 (90), 72.9934 (40), 133.0130 (24), 89.0239 (8)
Protocatechuic acid	153.0195	4.2	109.0299 (100), 123.0453 (74), 153.0189 (35), 153.0543 (20), 108.0187 (14), 110.032 (13)
Caftaric acid	311.0400	4.9	179.0354 (100), 149.0094 (50), 135.0454 (31)
Vanillin	151.0397	6.9	151.0414 (100), 109.0275 (21), 107.0502 (10), 108.4068 (8)
Caffeic acid	179.0349	7.3	135.0453 (100), 179.0355 (36), 136.0483 (10), 134.0369 (6), 180.0384 (5), 107.0501 (1)
Benzoic acid	121.0293	7.7	121.0287 (100), 122.0334 (8), 120.0232(5), 108.0246 (3)
Chicoric acid	473.0706	10.6	179.0349 (100), 149.0089 (96), 311.0389 (84), 293.0294 (30), 112.9874 (9), 135.0447 (7)
Rutin derivative	609.1458	11.0	609.1453 (100), 610.1492 (26) 300.0291 (21), 301.0344 (18), 611.1486 (9)

			**Ethanolic Extracts obtained from Roots**
Malic acid	133.0146	1.3	115.0042 (100), 71.0139 (62), 133.0156 (35), 72.9928 (22), 89.0246 (10), 43.0195 (8)
Caftaric acid	311.0419	4.9	179.0363 (100), 149.0105 (63), 135.0460 (23)
Chlorogenic acid	353.0882	6.4	191.0568 (100), 192.0589 (9), 353.0877 (4), 161.0243 (3), 179.0363 (1)
Quinic acid	191.0565	6.4	191.0566 (100), 102.9488 (16), 93.0336 (6)
Caffeic acid	179.0356	7.3	135.0456 (100), 179.0355 (48), 136.0493 (12), 134.0377 (9), 180.0397 (2), 107.0514 (1)
Benzoic acid	121.0296	7.7	121.0295 (100), 122.0330 (16), 108.0202 (6)
p-coumaric acid	163.0399	9.6	119.0506 (100), 163.0416 (22), 162.8389 (18), 120.0542 (9)
Chicoric acid	473.0716	10.6	149.0094 (100), 179.0351 (80), 311.0401 (57), 293.0300 (26), 135.0450 (10), 112.988 (8)
			**Dichloromethanolic Extracts obtained from Flowers**
Caffeic acid	179.0358	7.3	135.0447 (100), 179.0352 (68), 134.0397 (53), 180.9211 (24), 136.0509 (23)
Benzoic acid	121.0295	7.7	121.0295 (100), 122.0323 (15), 108.0234 (5), 120.0219 (4), 93.0358 (3)
Chicoric acid	473.0745	10.5	149.0106 (76), 179.0362 (58), 311.0409 (53)
			**Dichloromethanolic Extracts obtained from Leaves**
Malic acid	133.0145	1.3	115.0038 (100), 133.0151 (76), 71.0134 (73), 43.0191 (30), 89.0219 (29), 72.9927 (21)
Caftaric acid	311.0407	4.8	179.0350 (100), 149.0098 (80), 135.0442 (21)
Caffeic acid	179.0358	7.3	135.0449 (100), 179.0357 (26), 134.0381 (9), 136.8699 (8)
Benzoic acid	121.0292	7.7	121.0295 (100), 122.0330 (8), 108.0216 (5), 120.0237 (2), 121.2224 (2), 93.0341 (1)
Chicoric acid	473.0714	10.6	179.0356 (100), 149.0097 (94), 311.0410 (41), 293.0328 (13), 135.045 (5)
			**Dichloromethanolic Extracts obtained from Roots**
Malic acid	133.0140	1.3	115.0035 (100), 71.0139 (51), 133.014 (27), 72.9941(19), 89.0261 (15), 132.8685 (10)
Vanillic acid derivative	167.0347	6.6	152.0117 (100), 167.0356 (27), 152.2865 (2), 152.1520 (2), 108.0218 (2)
Caffeic acid	179.0351	7.3	135.0458 (100), 179.0352 (40), 134.0366 (5), 180.0359 (5), 107.0526 (4), 136.0465 (2)
Benzoic acid	121.0290	7.7	121.0296 (100), 122.0325 (9), 93.0334 (3), 108.0223 (3), 120.0218 (2)
p-coumaric acid	163.0403	9.5	119.0509 (100), 163.0405 (30), 162.8403 (18), 120.0524 (6)

[1] The MS^2 data were obtained from the fragmentation of the $[M - H]^-$ precursor ion of phenolic/acid compounds. Relative intensities of product ions are in parentheses.

Table S3. Alkylamides compounds tentatively identified in *E. purpurea* extracts by LC-HRMS (positive mode). F: flowers; L: leaves; R: roots; AE: aqueous extracts; EE: ethanolic extracts; DE: dichloromethanolic extracts.

Proposed alkylamide compound	Precursor ion [M + H]+ (m/z)	t_R (min)	Product ions (m/z) [1]
**Aqueous Extracts obtained from Flowers**			
Dodeca-2E,4Z,10E-triene-8-ynoic acid isobutylamide	246.1872	15.4	57.0702 (100), 128.0627 (27), 93.0712 (4), 145.1034 (3), 74.0963 (3), 173.0890 (2), 119.0876 (1), 190.5467 (0.1)
Dodeca-2E,4Z,10Z-triene-8-ynoic acid isobutylamide	246.1870	15.8	57.0702 (100), 128.0623 (26), 93.0710 (5), 173.0962 (2), 102.0913 (2), 119.0857 (1), 74.0384 (1)
Dodeca-2E,4E,10E-triene-8-ynoic acid isobutylamide	246.1860	20.1	57.0696 (100), 128.0618 (6), 119.0845 (2), 145.1025 (1), 74.0954 (1)
Undeca-2E,4Z-diene-8,10-diynoic acid isobutylamide	230.1551	20.2	57.0697 (100), 128.0622 (29), 91.0544 (23), 116.0618 (10), 129.0687 (8), 103.0539 (4), 117.0674 (2), 102.0910 (1), 74.0961 (1), 157.0652 (0.2), 174.0929 (0.1), 43.0541 (0.1)
Undeca-2E/Z-ene-8,10-diynoic acid isobutylamide	232.1705	20.4	57.0698 (100), 91.0543 (58), 41.0383 (38), 79.0538 (31), 65.0382 (20), 115.0541 (19), 105.0701 (17), 131.0843 (4), 74.0966 (0.8), 159.0816 (0.3), 176.1021 (0.3)
Dodeca-2E,4Z-diene-8,10-diynoic acid isobutylamide	244.1703	20.7	57.0695 (100), 43.0538 (31), 128.0623 (28), 105.0695 (6), 117.0686 (5), 143.0827 (2), 102.0930 (1), 39.0222 (1), 74.0944 (0.3)
Dodeca-2E-ene-8,10-diynoic acid isobutylamide	246.1857	20.9	81.0697 (1), 145.1011 (1), 105.0689 (1), 57.0693 (1), 74.0961 (1), 91.0549 (1), 173.0961 (1), 79.0542 (0.4), 67.0542 (0.3), 102.0553 (0.1), 190.1246 (0.2), 41.0365 (0), 77.0060 (0)
Trideca-2E,7Z-diene-10,12-diynoic acid isobutylamide	258.1861	20.9	57.0697 (100), 117.0699 (43), 128.0618 (32), 103.0540 (12), 131.0846 (4), 43.0541 (3), 157.1002 (2), 74.0969 (2), 202.1241 (0.4), 185.0984 (0.2)
Trideca-2E,7Z-diene-10,12-diynoic acid 2-methylbutylamide	272.2015	21.2	71.0846 (9), 157.1020 (9), 202.1231 (5)
Dodeca-2E,4E,8Z,10E/Z-tetraenoic acid isobutylamide	248.2031	21.2	57.0696 (100), 67.0539 (72), 68.0616 (52), 152.1072 (33), 81.0692 (26), 79.0536 (20), 41.038 (18), 66.0461 (17), 167.1307 (8), 100.0757 (6), 74.0967 (1), 147.1177 (1), 102.0909 (1), 168.1345 (1), 121.1010 (0.3), 109.1007 (0.3), 175.1121 (0.2), 142.1233 (0.2), 128.1077 (0.2), 192.4037 (0)

Dodeca-2E,4E,8Z-trienoic acid isobutylamide	250.2169	21.5	57.0696 (100), 168.1361 (1), 102.0909 (1), 194.1562 (0.4), 74.0962 (0.3), 177.1294 (0.1)
Dodeca-2E,4E,8Z,10E/Z-tetraenoic acid 2-methylbutylamide	262.2170	21.5	71.0858 (100), 147.1124 (3), 116.1099 (3)
Dodeca-2E,4E-dienoic acid isobutylamide	252.2323	22.0	57.0694 (100), 67.0540 (50), 55.0538 (50), 81.0688 (36), 69.0696 (31), 81.032 (23), 43.0541 (15), 95.0845 (11), 196.1707 (8), 154.1237 (2), 179.1433 (2), 168.1324 (2), 99.0653 (2), 74.0961 (1), 85.0643 (1), 151.1499 (1), 139.0968 (1)
**Aqueous Extracts obtained from Roots**			
Dodeca-2E,4Z,10E-triene-8-ynoic acid isobutylamide	246.1857	15.3	57.0690 (100), 128.0614 (6), 119.0854 (2), 102.0923 (2), 93.0696 (1)
Dodeca-2E,4Z,10Z-triene-8-ynoic acid isobutylamide	246.1859	15.8	57.0693 (100), 128.0608 (9), 145.1005 (4), 93.0697 (3), 154.1243 (1)
Dodeca-2E,4E,10Z-triene-8-ynoic acid isobutylamide	246.1853	19.0	57.0694 (100), 128.0618 (7), 119.0843 (2), 93.0700 (2), 190.1226 (1), 154.0629 (1), 145.1028 (1), 102.0465 (1), 74.0936 (1)
Dodeca-2Z,4E,10Z-triene-8-ynoic acid isobutylamide	246.1857	19.4	57.0697 (100), 128.0618 (15), 145.1013 (7), 173.0946 (2), 119.0884 (2), 102.0902 (2), 93.0610 (1), 74.0978 (1)
Undeca-2E,4Z-diene-8,10-diynoic acid isobutylamide	230.1548	20.2	57.0696 (100), 128.0619 (26), 91.054 (25), 116.0617 (10), 129.0687 (6), 103.0541 (3), 117.0650 (1), 102.0896 (1), 74.0961 (1), 174.0910 (0.3), 157.0669 (0.3), 43.0536 (0.3)
Undeca-2Z,4E-diene-8,10-diynoic acid isobutylamide	230.1561	20.5	57.0698 (100), 128.0623 (72), 91.0544 (49), 129.0691 (23), 103.0543 (7), 174.0909 (1), 157.0653 (1), 74.0968 (1), 102.0905 (0.4), 202.1197 (0.1)
Dodeca-2E,4Z-diene-8,10-diynoic acid isobutylamide	244.1712	20.6	57.0697 (100), 128.0621 (75), 91.0542 (20), 117.0701 (19), 105.0697 (17), 143.0857 (7), 39.0225 (4), 154.0657 (2), 43.0536 (2), 188.1083 (1), 171.0819 (1), 102.0914 (1), 74.0963 (1), 216.1393 (0.1)
Dodeca-2Z,4E-diene-8,10-diynoic acid isobutylamide	244.1708	20.9	57.0697 (100), 128.0621 (77), 105.0700 (33), 91.0542 (30), 117.0701 (26), 143.0859 (7), 39.0223 (5), 43.054 (5), 188.1076 (1), 171.0812 (1), 74.0967 (1), 216.137 (0.2), 102.0906 (0.2), 202.1635 (0.1), 154.1228 (0.1)
Trideca-2E,7Z-diene-10,12-diynoic acid isobutylamide	258.1856	21.0	43.0539 (100), 128.0621 (66), 117.0697 (21), 103.0539 (7), 57.0693 (7), 131.0800 (1), 74.0975 (0.4)

Dodeca-2,4-diene-8,10-diynoic acid 2-methylbutylamide	258.1856	21.0	71.0856 (64), 91.0540 (25),117.0697 (21), 105.0699 (20), 143.0857 (7), 57.0693 (7), 171.0806 (2), 188.1071 (1), 116.1053 (1), 88.1117 (0.4), 29.0374 (0.2), 230.1451 (0.1), 168.1363 (0.1)
*Dodeca-2Z,4Z,10Z-triene-8-ynoic acid isobutylamide**	246.1858	21.1	57.0697 (100), 128.0621 (23), 145.1013 (6), 173.0953 (2), 119.0862 (2), 93.0690 (2), 102.0904 (1), 74.0967 (1), 190.1215 (0.3)
Dodeca-2E,4E,8Z,10E/Z-tetraenoic acid isobutylamide	248.2023	21.2	57.0698 (100), 67.0541 (66), 68.0619 (44), 81.0694 (22), 152.1072 (21), 41.0383 (16), 79.0537 (15), 66.0462 (14), 167.1306 (5), 100.0756 (5), 102.091 (1), 74.0968 (1), 168.1348 (1), 147.1168 (0.3), 121.1018 (0.1), 128.1082 (0.1), 175.1107 (0), 192.1384 (0)
Dodeca-2E,4Z,10E-triene-8-ynoic acid 2-methylbutylamide OR Dodeca-2E-ene-8,10-diynoic acid 2-methylbutylamide	260.2018	21.2	71.0856 (40), 131.0867 (6), 145.1005 (5), 119.0852 (5), 190.1241 (1), 173.0948 (0.4), 88.1121 (0.4), 116.1222 (0.3)
Pentadeca-2E,9Z-diene-12,14-diynoic acid isobutylamide	286.2179	21.4	57.0699 (100), 74.0967 (16), 230.8572 (6), 213.1114 (6)
Dodeca-2E,4E,8Z-trienoic acid isobutylamide	250.2167	21.5	57.0697 (100), 194.1552 (1), 168.1350 (1), 149.1320 (1), 83.0840 (1), 74.0964 (1), 177.1256 (0.4), 97.1028 (0.4)
Dodeca-2E,4E-dienoic acid isobutylamide	252.2325	21.9	57.0697 (100), 55.0540 (57), 67.0541 (44), 69.0696 (31), 81.0334 (31), 81.0694 (25), 95.049 (13), 43.0538 (11), 196.1693 (5), 168.1348 (3), 74.0948 (3), 154.1229 (2), 179.1475 (1), 210.1826 (0.4), 139.1165 (0.4), 151.1470 (0.3), 102.0896 (0.3)
**Ethanolic Extracts obtained from Flowers**			
Dodeca-2E,4Z,10E-triene-8-ynoic acid isobutylamide	246.1853	15.4	57.0695 (100), 128.0617 (20), 145.1002 (13), 119.0864 (4), 74.0973 (4), 173.0958 (2), 93.0677 (2), 154.1263 (1), 190.0642 (0.4), 102.0782 (0.1)
Dodeca-2E,4Z,10Z-triene-8-ynoic acid isobutylamide	246.1851	15.8	57.0692 (100), 128.0615 (16), 145.1014 (4),119.0863 (3), 93.0674 (2), 173.0975 (1), 102.0908 (1), 74.0970 (1)
Dodeca-2E,4E,10Z-triene-8-ynoic acid isobutylamide	246.1857	19.0	57.0695 (100), 145.1013 (11), 128.0617 (9), 74.0969 (2), 119.0851 (1), 93.0697 (1), 190.1204 (0.4), 173.0881 (0.4)

Dodeca-2E,4E,10E-triene-8-ynoic acid isobutylamide	246.1854	20.1	57.0696 (41), 128.0618 (6), 119.0848 (3), 93.0691 (2), 145.1021 (1), 173.0958 (0.3), 74.0965 (0.3)
Undeca-2E,4Z-diene-8,10-diynoic acid isobutylamide	230.1571	20.2	57.0694 (100), 128.0618 (69), 91.0538 (33), 116.0614 (21), 129.0684 (15), 103.0536 (6), 117.0642 (3), 157.0653 (1), 102.0912 (1), 74.0967 (1), 174.0915 (0.3), 43.0534 (0.2)
Undeca-2E/Z-ene-8,10-diynoic acid isobutylamide	232.1698	20.4	57.0696 (100), 91.0541 (81), 41.0382 (49), 79.0537 (41), 105.0696 (32), 65.0383 (29), 115.0540 (28), 131.0851 (9), 176.1067 (1), 159.0802 (1), 102.0902 (0.3), 74.0966 (0.3)
Pentadeca-2E,9Z-diene-12,14-diynoic acid 2-hydroxyisobutylamide	302.2118	20.6	118.0746 (6), 73.0851 (4), 230.065 (1), 213.2401 (1), 185.1340 (1), 90.0554 (1)
Dodeca-2E,4Z-diene-8,10-diynoic acid isobutylamide	244.1704	20.7	57.0697 (83), 43.0539 (64), 128.062 (56), 91.0543 (30), 105.0701 (8), 117.0693 (7), 39.0226 (4), 143.0858 (2), 74.0970 (1), 171.0803 (0.2), 202.1285 (0.1), 188.1057 (0.1), 216.1477 (0), 142.1229 (0)
Dodeca-2E-ene-8,10-diynoic acid isobutylamide	246.1852	20.8	57.0696 (100), 67.0541 (63), 79.0538 (23), 91.0540 (21), 41.0383 (20), 105.0697 (13), 77.0385 (8), 81.0692 (5), 145.1015 (2), 190.1228 (1), 102.0914 (1), 74.0969 (1), 173.0962 (0.3)
Trideca-2E,7Z-diene-10,12-diynoic acid isobutylamide	258.1898	20.9	117.0698 (100), 57.0697 (61), 128.0617 (34), 131.0856 (32),157.1016 (16), 103.0538 (9), 202.1231 (6), 185.0966 (5), 74.0968 (2), 142.1198 (1), 43.0540 (1), 230.1522 (0.3), 216.1379 (0.3), 102.0916 (0)
Dodeca-2,4-diene-8,10-diynoic acid 2-methylbutylamide	258.1898	20.9	117.0698 (100), 57.0697 (61), 91.0540 (37), 143.0852 (17), 116.0599 (12), 202.1231 (6), 142.1198 (1), 171.0774 (0.4), 230.1522 (0.3), 188.1074 (0.1), 71.0858 (0.1), 29.0371 (0.1),
Dodeca-2E,4Z,10E-triene-8-ynoic acid 2-methylbutylamide OR Dodeca-2E-ene-8,10-diynoic acid 2-methylbutylamide	260.2001	21.0	116.0604 (20), 145.1016 (7), 71.0857 (7), 119.0853 (5), 142.1229 (0.3), 173.0810 (0.1)
Trideca-2E,7Z-diene-10,12-diynoic acid 2-methylbutylamide	272.2009	21.1	71.0859 (28), 157.1015 (2), 202.1229 (1), 185.0991 (1), 88.1128 (1)
Dodeca-2E,4E,8Z,10E/Z-tetraenoic acid isobutylamide	248.2041	21.3	57.0699 (100), 67.0541 (92), 152.108 (90), 68.0617 (74), 81.0692 (45), 79.0534 (36), 41.0379 (35), 66.0457 (33), 167.1318 (26), 100.0762 (9), 168.1352 (3), 102.0918 (3), 74.0968 (2), 147.1178 (2), 109.1012 (1), 128.1079 (1), 121.1014 (1), 175.113 (1), 142.1238 (0.2),

Dodeca-2E,4E,8Z-trienoic acid isobutylamide (isomer 1)	250.2146	21.4	57.0697 (100), 83.0357 (3), 169.1346 (2), 102.0908 (2), 154.1196 (1), 74.0959 (1), 149.1336 (0.4), 194.1543 (0.1), 177.1261 (0.1), 97.1015 (0.1)
Pentadeca-2E,9Z-diene-12,14-diynoic acid isobutylamide	286.2160	21.4	57.0697 (99), 74.097 (2), 230.1521 (1), 185.1325 (1)
Dodeca-2E,4E,8Z-trienoic acid isobutylamide	250.2173	21.5	57.0696 (100), 168.135 (2), 102.0911 (2), 83.0358 (2), 74.0970 (2), 194.1540 (1), 177.1272 (1), 154.1219 (1), 149.1336 (1)
Dodeca-2E,4E,8Z,10E/Z-tetraenoic acid 2-methylbutylamide	262.2165	21.5	71.0855 (100), 116.1071 (2), 147.1175 (1), 88.1122 (1), 192.1400 (0.3), 175.1121 (0.2)
Dodeca-2E,4E-dienoic acid isobutylamide	252.2361	21.9	57.0696 (100), 55.0539 (86), 81.0328 (77), 67.0539 (54), 69.0697 (53), 81.0695 (46), 95.0855 (31), 196.1706 (21), 43.0537 (15), 154.1234 (7), 179.1437 (3), 168.1387 (3), 74.0971 (3), 151.1492 (2), 85.1013 (0.4), 210.1858 (0.3), 116.1071 (0.2), 102.0909 (0.2)
**Ethanolic Extracts obtained from Leaves**			
Dodeca-2E,4E,10E-triene-8-ynoic acid isobutylamide	246.1860	20.0	57.0695 (100), 128.0615 (12), 119.0855 (3), 93.0695 (3), 173.0814 (1), 145.1023 (2), 102.0878 (1), 74.0967 (1)
Undeca-2E,4Z-diene-8,10-diynoic acid isobutylamide	230.1540	20.2	57.0699 (100), 91.0538 (28), 128.0617 (25), 129.0690 (7), 116.0626 (7), 43.0539 (6), 103.0536 (4), 117.0683 (2), 202.0911 (1), 157.0989 (1), 102.0906 (1), 188.1023 (0.3)
Pentadeca-2E,9Z-diene-12,14-diynoic acid 2-hydroxyisobutylamide	302.2113	20.6	118.0742 (5), 185.1314 (3), 213.1242 (1), 73.0647 (1), 90.0923 (0.2)
Dodeca-2E-ene-8,10-diynoic acid isobutylamide	246.1850	20.9	57.0694 (100), 67.0538 (52), 41.0380 (19), 79.0535 (13), 81.0689 (11), 105.0694 (6), 91.0535 (5), 77.0394 (3), 173.0918 (1), 145.0989 (1), 102.0913 (1), 74.0975 (1)
Dodeca-2E,4E,8Z,10E/Z-tetraenoic acid isobutylamide	248.2010	21.2	57.0696 (100), 67.0539 (51), 68.0617 (32), 152.1066 (16), 41.0382 (13), 81.0691 (13), 79.0536 (12), 66.0463 (10), 109.1012 (1), 102.0911 (1), 175.1108 (0.3), 168.1313 (0.3), 192.1359 (0.2), 147.1214 (0.2), 142.1261 (0.2), 74.0955 (0.2),
Dodeca-2E,4E-dienoic acid isobutylamide (isomer 1)	252.2321	21.4	57.0694 (30), 168.1338 (1), 85.1011 (1), 194.1705 (0.4), 139.1418 (0.4), 116.0625 (0.4), 74.0969 (0.4), 102.0912 (0.3)

Pentadeca-2E,9Z-diene-12,14-diynoic acid isobutylamide	286.2165	21.4	57.0695 (99), 74.0947 (5), 185.1301 (3), 230.1547 (1),
Dodeca-2E,4E-dienoic acid isobutylamide	252.2317	21.9	57.0694 (100), 55.0538 (53), 67.0537 (52), 69.0696 (30), 81.0687 (28), 81.0328 (25), 95.0848 (9), 196.1683 (8), 43.0535 (8), 154.1222 (2), 179.1426 (1), 151.151 (1), 102.0928 (1), 74.0979 (1)
**Ethanolic Extracts obtained from Roots**			
Dodeca-2E,4Z,10E-triene-8-ynoic acid isobutylamide	246.1881	15.3	57.0707 (100), 128.0629 (14), 93.0723 (3), 145.1034 (2), 119.0879 (1)
Dodeca-2E,4Z,10Z-triene-8-ynoic acid isobutylamide	246.1872	15.8	57.0704 (100), 128.0636 (14), 119.0856 (2), 154.1251 (1), 74.0973 (1)
Dodeca-2E,4E,10Z-triene-8-ynoic acid isobutylamide	246.1869	19.0	57.0702 (100), 128.0633 (9), 145.1023 (4), 119.0849 (3), 102.0928 (3), 93.0694 (3), 173.0944 (1), 74.0960 (1), 190.1292 (0.3)
Dodeca-2Z,4E,10Z-triene-8-ynoic acid isobutylamide	246.1869	19.4	57.0699 (100), 145.1016 (11), 128.0613 (10), 93.0695 (3), 173.0976 (2), 119.0861 (2), 190.1227 (1), 74.0888 (1)
Dodeca-2E,4E,10E-triene-8-ynoic acid isobutylamide	246.1863	20.1	57.0698 (100), 128.0624 (15), 93.0703 (2), 173.0286 (1), 154.1231 (1), 119.0867 (1), 102.0906 (1), 145.0977 (0.3)
Undeca-2E,4Z-diene-8,10-diynoic acid isobutylamide	230.1586	20.2	57.0703 (100), 128.0628 (64), 91.0549 (34), 129.0695 (16), 103.0548 (6), 102.0921 (1), 74.0977 (1), 174.0920 (0.4), 157.0667 (0.4), 43.0541 (0.2)
Undeca-2Z,4E-diene-8,10-diynoic acid isobutylamide	230.1580	20.5	129.0702 (100), 128.0625 (88), 57.0701 (85), 91.0545 (64), 116.0624 (51), 157.0661 (15), 174.0928 (13), 103.0544 (10), 117.0656 (7), 202.1241 (1), 102.0915 (1), 74.0972 (1), 188.1078 (0.2), 43.0546 (0.2)
Dodeca-2E,4Z-diene-8,10-diynoic acid isobutylamide	244.1737	20.7	128.0626 (100), 57.0700 (66), 117.0704 (22), 91.0543 (22), 105.0702 (18), 143.0865 (10), 43.0541 (5), 39.0227 (5), 171.0816 (2), 188.1089 (1), 102.0915 (1), 74.0970 (1), 216.1401 (0.1), 154.1200 (0.1), 202.1225 (0), 142.1286 (0)
Undeca-2E,4E-diene-8,10-diynoic acid isobutylamide	230.1552	20.8	57.0701 (100), 128.0628 (52), 91.0547 (29), 129.0696 (15), 116.0620 (13), 103.0544 (5), 117.0603 (4), 174.0927 (1), 157.0661 (1), 102.0927 (0.3), 74.0980 (0.2), 43.0551 (0.1)
Dodeca-2Z,4E-diene-8,10-diynoic acid isobutylamide	244.1729	20.9	128.0623 (100), 117.0701 (86), 57.0699 (84), 105.0700 (67), 143.0860 (47), 91.0541 (25), 171.0811 (17), 188.1077 (7), 43.0540 (7), 74.0966 (2), 39.0228 (2), 102.0907 (0.4), 154.1219 (0.2), 142.1228 (0.2), 202.1238 (0.1), 206.1501 (0)

Dodeca-2E-ene-8,10-diynoic acid isobutylamide	246.1857	20.9	57.0699 (78), 67.0542 (43), 105.0701 (35), 41.0384 (34), 91.0542 (33), 79.0540 (30), 77.0385 (19), 145.1016 (6), 81.0696 (4), 173.0960 (1), 74.0969 (1), 102.0912 (0.3), 190.1233 (0.2)
Trideca-2E,7Z-diene-10,12-diynoic acid isobutylamide	258.1900	21.0	117.0702 (100), 128.0624 (96), 43.0542 (61), 131.0838 (9), 57.0698 (8), 103.0545 (7), 157.1016 (2), 230.1553 (2), 216.1752 (1), 202.1235 (1), 142.1236 (1), 74.0969 (1), 185.0962 (0.4), 156.1364 (0.1)
Dodeca-2,4-diene-8,10-diynoic acid 2-methylbutylamide	258.1900	21.0	117.0702 (100), 143.0861 (75), 71.0859 (66), 105.0701 (42), 91.0544 (24), 171.0814 (23), 188.1079 (22), 116.0608 (8), 57.0698 (8), 230.1553 (2), 88.1124 (2), 202.1235 (1), 142.1236 (1), 168.1379 (0.2), 156.1364 (0.1), 29.0390 (0.1)
Dodeca-2Z,4Z,10Z-triene-8-ynoic acid isobutylamide [2]	246.1862	21.1	57.0701 (100), 128.0626 (50), 145.1021 (15), 119.0864 (12), 173.0969 (4), 93.0704 (4), 74.0969 (2), 190.1238 (1), 102.0915 (1), 154.122 (0.2)
Trideca-2E,7Z-diene-10,12-diynoic acid 2-methylbutylamide	272.2018	21.1	71.0858 (21), 157.1012 (3), 202.1228 (1), 185.0954 (1), 88.1118 (1), 116.1077 (0.2)
Dodeca-2E,4E,8Z,10E/Z-tetraenoic acid isobutylamide	248.2048	21.2	57.0702 (100), 67.0544 (91), 152.1085 (85), 68.0621 (75), 81.0697 (44), 79.0538 (36), 41.0383 (32), 66.0462 (31), 167.1324 (23), 100.0769 (10), 168.1360 (3), 102.0922 (3), 74.0974 (2), 147.1186 (1), 128.1086 (1), 121.1019 (1), 109.1021 (1), 192.1407 (0.3), 175.1132 (0.3), 142.1253 (0.2)
Dodeca-2E,4Z,10E-triene-8-ynoic acid 2-methylbutylamide OR Dodeca-2E-ene-8,10-diynoic acid 2-methylbutylamide	260.2017	21.2	71.086 (48), 131.0861 (14), 145.1019 (13), 119.0862 (13), 173.0960 (2), 116.1079 (1), 88.1123 (1), 190.1243 (0.4), 232.2067 (0.2)
Pentadeca-2E,9Z-diene-12,14-diynoic acid isobutylamide	286.2170	21.4	57.0701 (80), 74.0969 (2), 185.1354 (1), 230.1573 (0.2), 213.1271 (0.2)
Dodeca-2E,4E,8Z,10E/Z-tetraenoic acid 2-methylbutylamide	262.2177	21.5	71.0859 (100), 116.1073 (3), 147.1178 (1), 145.1019 (1), 88.1117 (1), 175.1126 (0.3), 192.1368 (0.1)
Trideca-2Z,7Z-diene-10,12-diynoic acid 2-methylbutylamide	272.2011	21.5	71.0862 (2), 157.1012 (2), 185.0929 (0.4), 202.1644 (0.3)
Dodeca-2E,4E,8Z-trienoic acid isobutylamide	250.2180	21.6	57.0700 (100), 83.0856 (3), 168.1353 (2), 102.092 (2), 74.0973 (2), 194.1547 (1), 177.1283 (1), 154.1233 (1), 149.1336 (0.4), 97.1017 (0.2)

Hexadeca-2E,9Z-diene-12,14-diynoic acid isobutylamide	300.2323	21.6	57.0699 (74), 74.0959 (6), 227.1462 (1), 199.1527 (1)
Dodeca-2E,4E-dienoic acid isobutylamide	252.2359	21.9	57.0698 (100), 55.0541 (85), 81.0331 (77), 67.0542 (53), 69.0700 (50), 81.0698 (44), 95.0858 (31), 196.1711 (17), 43.0541 (16), 154.1235 (6), 179.1443 (3), 74.0972 (3), 168.1392 (2), 151.1491 (1), 85.1014 (1), 116.1084 (0.4), 210.1864 (0.2), 102.0909 (0.1)
**Dichloromethanolic Extracts obtained from Flowers**			
Dodeca-2E,4Z,10E-triene-8-ynoic acid isobutylamide	246.1857	15.3	57.0694 (100), 128.0603 (11), 146.0957 (3), 119.0840 (2), 173.0132 (2), 154.1240 (2), 93.0551 (2), 102.0267 (1), 74.0526 (1)
Dodeca-2E,4Z,10Z-triene-8-ynoic acid isobutylamide2	246.1854	15.9	57.0692 (100), 145.1012 (8), 128.0607 (8), 93.0689 (4), 119.0840 (3), 74.0976 (3), 173.0775 (1), 102.0902 (1)
Dodeca-2,4,10-triene-8-ynoic acid isobutylamide (isomer 1)	246.1856	18.2	57.0694 (100), 128.0614 (19), 119.0852 (9), 145.1013 (6), 93.0700 (3), 102.0919 (2), 173.0952 (1), 154.1236 (1), 74.0967 (1),
Dodeca-2E,4E,10Z-triene-8-ynoic acid isobutylamide	246.1856	19.0	57.0695 (100), 145.1014 (17), 128.0616 (9), 119.0851 (4), 94.0695 (3), 74.0967 (3), 190.1229 (2), 173.0601 (1), 102.0922 (1)
Dodeca-2E,4E,10E-triene-8-ynoic acid isobutylamide	246.1854	20.1	57.0699 (9), 119.0851 (3), 128.0632 (2), 93.0694 (2), 145.0997 (1), 173.0977 (0.3)
Undeca-2E,4Z-diene-8,10-diynoic acid isobutylamide	230.1573	20.2	57.0696 (100), 128.0622 (85), 91.0540 (37), 116.0619 (26), 129.0687 (20), 103.054 (7), 117.0643 (3), 174.0919 (1), 157.0657 (1), 102.0914 (1), 74.0964 (1), 43.0536 (0.3)
Undeca-2E/Z-ene-8,10-diynoic acid isobutylamide	232.1700	20.4	57.0697 (100), 91.0542 (84), 41.0382 (47), 79.0539 (41), 115.0543 (34), 105.0700 (32), 65.0384 (27), 131.0857 (9), 176.1077 (2), 159.0806 (1), 74.0964 (1), 102.0916 (0.4)
Pentadeca-2E,9Z-diene-12,14-diynoic acid 2-hydroxyisobutylamide	302.2118	20.6	213.1258 (2), 185.0949 (2), 118.0728 (2), 230.1468 (1), 90.0898 (1)
Dodeca-2E,4Z-diene-8,10-diynoic acid isobutylamide	244.1714	20.7	128.062 (71), 57.0696 (80), 43.0538 (68), 91.0539 (30), 117.0695 (10), 105.0697 (8), 39.0224 (4), 143.0853 (3), 102.0911 (1), 74.0969 (1), 188.1061 (0.3), 171.0809 (0.3)
Dodeca-2E-ene-8,10-diynoic acid isobutylamide	246.1852	20.8	57.0698 (100), 67.0541 (66), 79.0538 (24), 91.0541 (23), 41.0382 (20), 105.0697 (14), 77.0385 (9), 81.0696 (6), 145.1012 (2), 190.1219 (1), 102.0909 (1), 74.0966 (1), 173.0938 (0.3)

Trideca-2E,7Z-diene-10,12-diynoic acid isobutylamide	258.1893	20.9	117.0698 (59), 128.0619 (57), 57.0695 (54), 103.0539 (16), 131.0857 (7), 157.1016 (2), 43.0538 (2), 202.1241 (1), 185.0973 (1), 74.0971 (1), 142.1203 (0.1), 230.1516 (0), 216.1391 (0), 102.0908 (0)
Dodeca-2,4-diene-8,10-diynoic acid 2-methylbutylamide	258.1893	20.9	117.0698 (59), 91.0538 (56), 57.0695 (54), 105.0697 (21), 143.0844 (5), 202.1241 (1), 29.0380 (0.3), 171.0726 (0.1), 142.1203 (0.1), 230.1516 (0), 188.1025 (0), 156.1364 (0), 88.1078 (0)
Dodeca-2E,4E,8Z,10E/Z-tetraenoic acid isobutylamide	248.2041	21.2	57.0699 (100), 67.0541 (92), 152.1077 (91), 68.0618 (74), 81.0691 (44), 41.0380 (37), 79.0534 (36), 66.0457 (33), 167.1315 (27), 100.0761 (10), 168.1350 (3), 102.0914 (3), 74.0967 (2), 175.1131 (1), 147.1176 (1), 128.1079 (1), 121.1020 (1), 109.1009 (1), 192.1382 (0.3), 142.1229 (0.3)
Dodeca-2E,4Z,10E-triene-8-ynoic acid 2-methylbutylamide OR Dodeca-2E-ene-8,10-diynoic acid 2-methylbutylamide	260.2006	21.0	116.0602 (21), 119.0853 (5), 71.0857 (5), 145.1015 (8), 142.1219 (0.4), 190.1213 (0.1), 173.0857 (0.1), 88.1141 (0)
Trideca-2E,7Z-diene-10,12-diynoic acid 2-methylbutylamide	272.2009	21.1	71.0858 (27), 157.1022 (2), 202.1235 (1), 185.0945 (1), 88.1120 (1), 116.1024 (0.1)
Dodeca-2E,4E,8Z-trienoic acid isobutylamide (isomer 1)	250.2140	21.4	57.0697 (100), 158.1348 (2), 102.0915 (2), 74.0967 (2), 177.1283 (1), 154.1205 (1), 149.1328 (1), 83.0857 (0.4), 194.1552 (0.3), 97.0996 (0.1)
Pentadeca-2E,9Z-diene-12,14-diynoic acid isobutylamide	286.2154	21.4	57.0696 (93), 185.1319 (2), 230.1569 (1), 213.1262 (1), 74.0976 (1)
Dodeca-2E,4E,8Z-trienoic acid isobutylamide	250.2184	21.5	57.0697 (100), 168.1356 (3), 102.0917 (3), 83.0358 (3), 154.1223 (2), 74.0964 (2), 194.1550 (1), 177.1275 (1), 149.1338 (1)
Dodeca-2E,4E,8Z,10E/Z-tetraenoic acid 2-methylbutylamide	262.2168	21.5	71.0856 (100), 116.1075 (3), 147.1171 (1), 88.1124 (1), 175.1130 (0.4), 192.1386 (0.3)
Dodeca-2E,4E-dienoic acid isobutylamide	252.2366	21.9	57.0695 (100), 55.0538 (86), 81.0328 (81), 67.0538 (57), 69.0697 (52), 81.0694 (47), 95.0853 (32), 196.1708 (20), 43.0537 (15), 154.1234 (8), 179.1442 (4), 74.0968 (3), 168.1394 (2), 151.1489 (2), 85.1012 (1), 210.1865 (0.4), 116.1069 (0.2), 102.0913 (0.1),
**Dichloromethanolic Extracts obtained from Leaves**			

Dodeca-2E,4E,10E-triene-8-ynoic acid isobutylamide	246.1851	20.1	57.0694 (100), 119.0863 (6), 102.0900 (2), 190.1682 (1), 145.1028 (1)
Undeca-2E,4Z-diene-8,10-diynoic acid isobutylamide	230.1542	20.2	57.0696 (100), 91.0544 (23), 128.0618 (17), 129.0679 (5), 43.0539 (4), 103.0524 (2), 157.0626 (1), 102.0889 (1), 74.0966 (1)
Pentadeca-2E,9Z-diene-12,14-diynoic acid 2-hydroxyisobutylamide	302.2116	20.6	118.0730 (3), 185.1330 (2), 90.0924 (1), 213.1278 (0.4), 73.0659 (0.1)
Dodeca-2E,4Z-diene-8,10-diynoic acid isobutylamide	244.1695	20.7	57.0696 (100), 43.0539 (57), 128.0624 (32), 91.0539 (21), 105.0696 (8), 117.0667 (6), 143.0854 (2), 74.0963 (2), 39.0209 (2), 188.1373 (1), 171.0779 (1), 154.1102 (1),
Dodeca-2E-ene-8,10-diynoic acid isobutylamide	246.1853	20.9	57.0695 (100), 67.0542 (73), 41.0383 (17), 91.0538 (13), 79.0534 (12), 81.0685 (8), 105.0694 (6), 145.0991 (1), 102.0940 (1)
Dodeca-2E,4E,8Z,10E/Z-tetraenoic acid isobutylamide	248.2009	21.3	57.0699 (100), 67.0543 (48), 68.0620 (35), 81.0697 (15), 79.0541 (13), 152.1068 (12), 41.0382 (10), 66.0464 (8), 167.1307 (5), 100.0762 (3), 168.1346 (1), 102.0920 (1), 74.0971 (1), 175. (0.2), 147.1186 (0.2), 192.1339 (0.1), 128.1075 (0.1)
Pentadeca-2E,9Z-diene-12,14-diynoic acid isobutylamide	286.2159	21.4	57.0694 (100), 185.133 (2), 230.1276 (1), 213.1644 (1), 74.0967 (1)
Dodeca-2E,4E-dienoic acid isobutylamide	252.2320	22.0	57.0696 (100), 55.0539 (70), 67.0540 (56), 81.0329 (38), 81.0695 (32), 69.0696 (32), 43.0539 (19), 95.0494 (17), 196.1698 (6), 154.1221 (3), 74.0982 (2), 179.1463 (1), 168.1379 (1), 151.1449 (1)
**Dichloromethanolic Extracts obtained from Roots**			
Dodeca-2E,4Z,10E-triene-8-ynoic acid isobutylamide	246.1855	15.4	57.0693 (100), 119.0855 (16), 128.062 (11), 145.0994 (10), 173.0957 (4), 93.0673 (3), 190.1252 (2), 154.1219 (1), 74.0933 (1)
Dodeca-2E,4Z,10Z-triene-8-ynoic acid isobutylamide	246.1856	15.8	57.0693 (100), 119.0866 (15), 145.1014 (12), 128.062 (8), 93.0697 (4), 74.1008 (2), 190.1167 (2), 173.0994 (1)
Dodeca-2,4,10-triene-8-ynoic acid isobutylamide (isomer 1)	246.1849	18.2	57.0694 (100), 128.0610 (20), 145.1003 (9), 119.0842 (9), 93.0689 (4), 173.0964 (3), 74.0964 (3), 190.1222 (1),
Dodeca-2E,4E,10Z-triene-8-ynoic acid isobutylamide	246.1849	19.0	57.0694 (100), 128.0612 (22), 145.1005 (12), 119.0849 (8), 93.0691 (5), 74.0958 (3), 173.0941 (1), 154.1244 (1), 190.1216 (0.4), 102.0913 (0.3)

Dodeca-2Z,4E,10Z-triene-8-ynoic acid isobutylamide	246.1845	19.4	57.0693 (100), 145.1005 (19), 119.0851 (16), 128.0617 (14), 173.0959 (4), 93.0692 (4), 74.0955 (2), 190.1211 (1)
Dodeca-2E,4E,10E-triene-8-ynoic acid isobutylamide	246.1850	20.1	57.0693 (48), 128.0601 (4), 119.0847 (3), 93.0701 (3), 190.1251 (1), 145.1013 (1), 74.0952 (1)
Undeca-2E,4Z-diene-8,10-diynoic acid isobutylamide	230.1566	20.2	128.0616 (100), 57.0693 (80), 91.0535 (42), 116.0612 (28), 129.0682 (26), 103.0533 (7), 117.0645 (4), 174.0921 (1), 157.0649 (1), 102.0909 (1), 74.0961 (1), 188.1425 (0.1), 43.0527 (0.1)
Undeca-2Z,4E-diene-8,10-diynoic acid isobutylamide	230.1560	20.6	129.0691 (100), 128.0614 (88), 91.0536 (54), 174.0913 (14), 57.0694 (62), 157.0647 (17), 103.0533 (10), 117.0645 (8), 202.1223 (1), 102.0901 (1), 74.0959 (1), 43.0529 (0.2), 188.1054 (0.1)
Dodeca-2E,4Z-diene-8,10-diynoic acid isobutylamide	244.1719	20.6	128.0616 (100), 57.0693 (57), 117.0692 (24), 91.0532 (22), 105.0691 (20), 143.0850 (12), 39.0220 (5), 188.1074 (2), 171.0802 (2), 43.0535 (2), 102.0910 (1), 74.0966 (1), 216.1396 (0.2), 142.1233 (0), 202.1253 (0)
Undeca-2E,4E-diene-8,10-diynoic acid isobutylamide	230.1535	20.8	57.0696 (100), 128.0618 (72), 91.0543 (45), 116.0617 (21), 129.0684 (20), 103.0540 (7), 117.0614 (3), 174.0904 (1), 157.0660 (1), 74.0966 (1), 102.0921 (0.4), 43.0535 (0.3), 202.1225 (0.2)
Dodeca-2E-ene-8,10-diynoic acid isobutylamide	246.1848	20.9	57.0692 (65), 67.0534 (39), 105.0691 (39), 91.0533 (35), 41.0378 (33), 79.0531 (28), 77.0376 (19), 145.1007 (7), 81.0687 (4), 173.0944 (1), 74.0959 (1), 190.1224 (0.3), 102.0903 (0.4)
Trideca-2E,7Z-diene-10,12-diynoic acid isobutylamide	258.1886	21.0	117.0693 (100), 128.0614 (95), 43.0537 (57), 131.0822 (8), 103.0533 (8), 57.0693 (5), 230.1542 (2), 157.0999 (1), 142.1220 (1), 74.0962 (1), 202.1230 (0.4), 185.0950 (0.2), 216.1385 (0.1), 156.1356 (0.1)
Dodeca-2,4-diene-8,10-diynoic acid 2-methylbutylamide	258.1886	21.0	117.0693 (100), 143.0851 (77), 71.0852 (60), 105.0691 (42), 188.1068 (24), 171.0801 (24), 91.0536 (22), 57.0699 (5), 230.1542 (2), 88.1116 (2), 142.1220 (1), 116.1062 (1), 202.1230 (0.4), 168.1360 (0.3), 156.1356 (0.1), 29.0383 (0)
*Dodeca-2Z,4Z,10Z-triene-8-ynoic acid isobutylamide**	246.1850	21.1	57.0692 (100), 128.0613 (67), 145.1008 (21), 119.0847 (13), 173.0958 (4), 93.0688 (4), 74.0962 (2), 190.1218 (1), 154.1216 (0.3), 102.0900 (0.3)
Dodeca-2E,4E,8Z,10E/Z-tetraenoic acid isobutylamide	248.2036	21.2	57.0695 (100), 67.0537 (93), 152.1072 (84), 68.0614 (76), 81.0688 (45), 79.0530 (37), 41.0377 (34), 66.0454 (32), 167.1310 (25), 100.0755 (9), 55.0533 (6), 168.1346 (3), 102.0911 (3), 81.0324 (2), 147.1174 (1), 128.1070 (1), 121.1003 (1), 109.1011 (1), 175.1122 (0.4), 142.1222 (0.3), 192.1383 (0.2)
Trideca-2E,7Z-diene-10,12-diynoic acid 2-methylbutylamide	272.1998	21.1	71.0852 (22), 157.1000 (2), 202.1211 (1), 185.0960 (1), 88.1101 (0.4), 116.1055 (0.1)

Dodeca-2E,4Z,10E-triene-8-ynoic acid 2-methylbutylamide OR Dodeca-2E-ene-8,10-diynoic acid 2-methylbutylamide	260.2005	21.2	71.0856 (48), 145.1011 (18), 131.0850 (18), 119.0852 (17), 57.0696 (3), 173.0960 (2), 190.1225 (1), 116.1080 (1), 88.1121 (1), 232.2059 (0.3)
Pentadeca-2E,9Z-diene-12,14-diynoic acid isobutylamide	286.2154	21.4	57.0695 (85), 74.0962 (2), 230.1546 (1), 185.1326 (1), 213.1272 (0.3), 102.0924 (0.1)
Dodeca-2E,4E,8Z-trienoic acid isobutylamide	250.2179	21.5	57.0694 (100), 168.1351 (3), 102.0908 (3), 83.0848 (3), 154.1221 (2), 74.0960 (2), 194.1546 (1), 177.1277 (1), 149.1330 (1), 97.1008 (0.2)
Dodeca-2E,4E,8Z,10E/Z-tetraenoic acid 2-methylbutylamide	262.2171	21.5	71.0855 (100), 116.1071 (3), 175.1113 (1), 147.1176 (1), 88.1118 (1), 192.1395 (0.3)
Trideca-2Z,7Z-diene-10,12-diynoic acid 2-methylbutylamide	272.1997	21.5	116.0610 (8), 157.0644 (4), 202.1258 (0.5), 71.0873 (0.4), 185.0925 (0.3)
Hexadeca-2E,9Z-diene-12,14-diynoic acid isobutylamide	300.2310	21.6	57.0695 (58), 74.0962 (5), 199.1484 (3), 227.1580 (1), 102.0417 (1)
Dodeca-2E,4E,8Z-trienoic acid isobutylamide (isómer 2)	250.2160	21.8	57.0696 (100), 83.0852 (2), 74.0964 (2), 194.1545 (1), 177.1274 (1), 168.1356 (1), 154.1225 (1), 102.0906 (1), 149.1323 (0.3)
Dodeca-2E,4E-dienoic acid isobutylamide	252.2360	21.9	57.0695 (100), 55.0538 (87), 81.0328 (76), 67.0538 (56), 69.0697 (53), 81.0695 (44), 95.0856 (31), 196.1704 (19), 43.0537 (15), 154.1234 (7), 179.1443 (3), 74.0971 (3), 168.1388 (2), 151.1486 (2), 85.1013 (1), 210.1854 (0.3), 116.1085 (0.2), 102.0907 (0.2)

[1] The MS^2 data were obtained from the fragmentation of the $[M + H]^+$ precursor ion of phenolic/acid compounds. Relative intensities of product ions are in parentheses.

[2] This compound was not found in literature.

E/Z stereochemistry is indicated here in accordance with literature [1–7], but it should be highlighted that without conformational NMR spectra, it is not possible to conclusively distinguish between E and Z isomers.

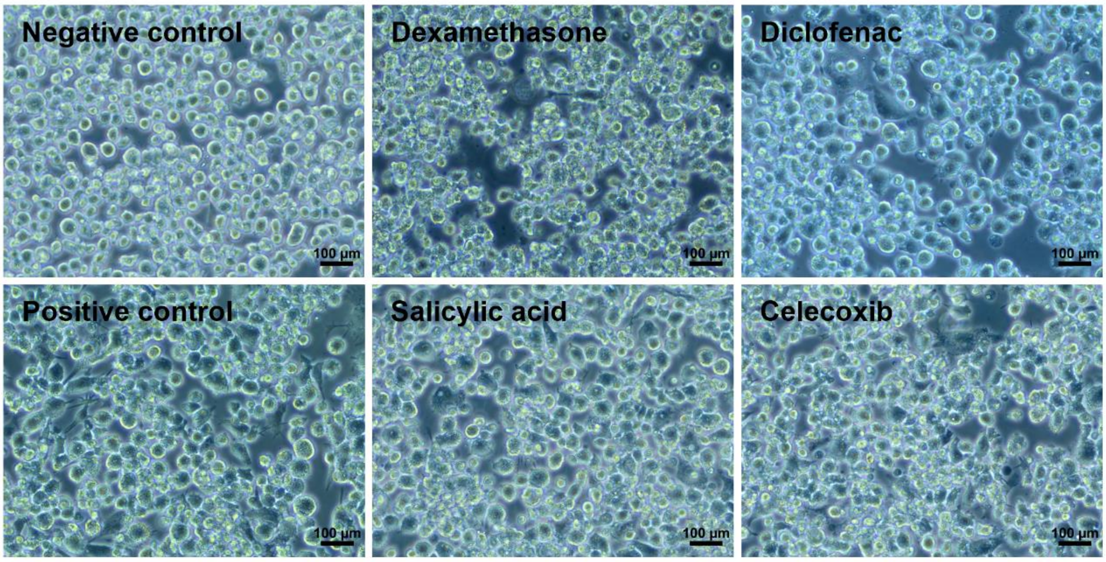

Figure S1. Optical micrographs of non-stimulated macrophages (negative control), LPS-stimulated macrophages (positive control) and LPS-stimulated macrophages cultured in the presence of clinically used anti-inflammatory drugs (dexamethasone, diclofenac, salicylic acid and celecoxib, 10 µM) cultured for 24 h. Scale bar = 100 µm.

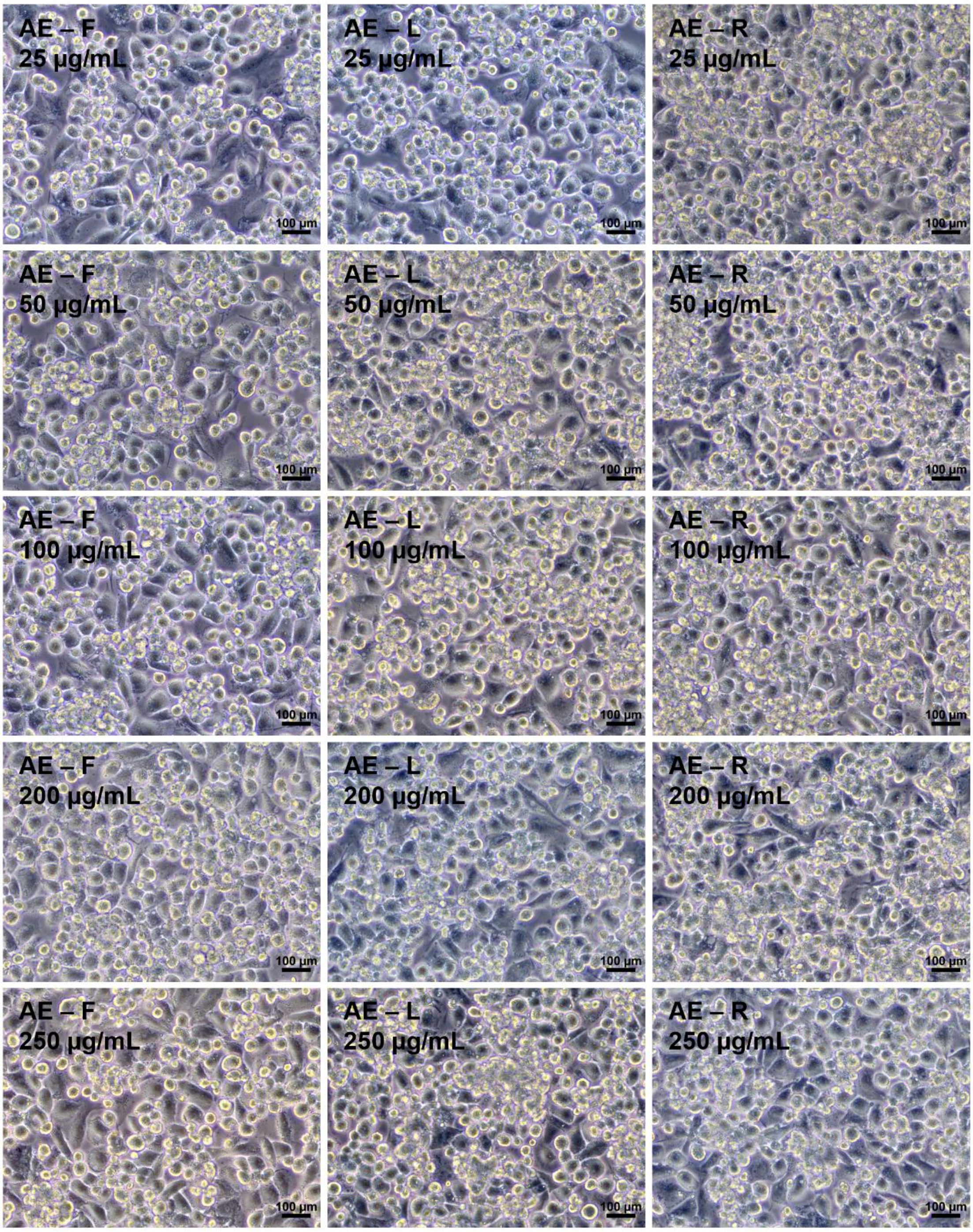

Figure S2. Optical micrographs of non-stimulated macrophages cultured in the presence of aqueous extracts (AE; 25 - 250 µg/mL) obtained from *E. purpurea* flowers (F), leaves (L), and roots (R) cultured for 24 h. Scale bar = 100 µm.

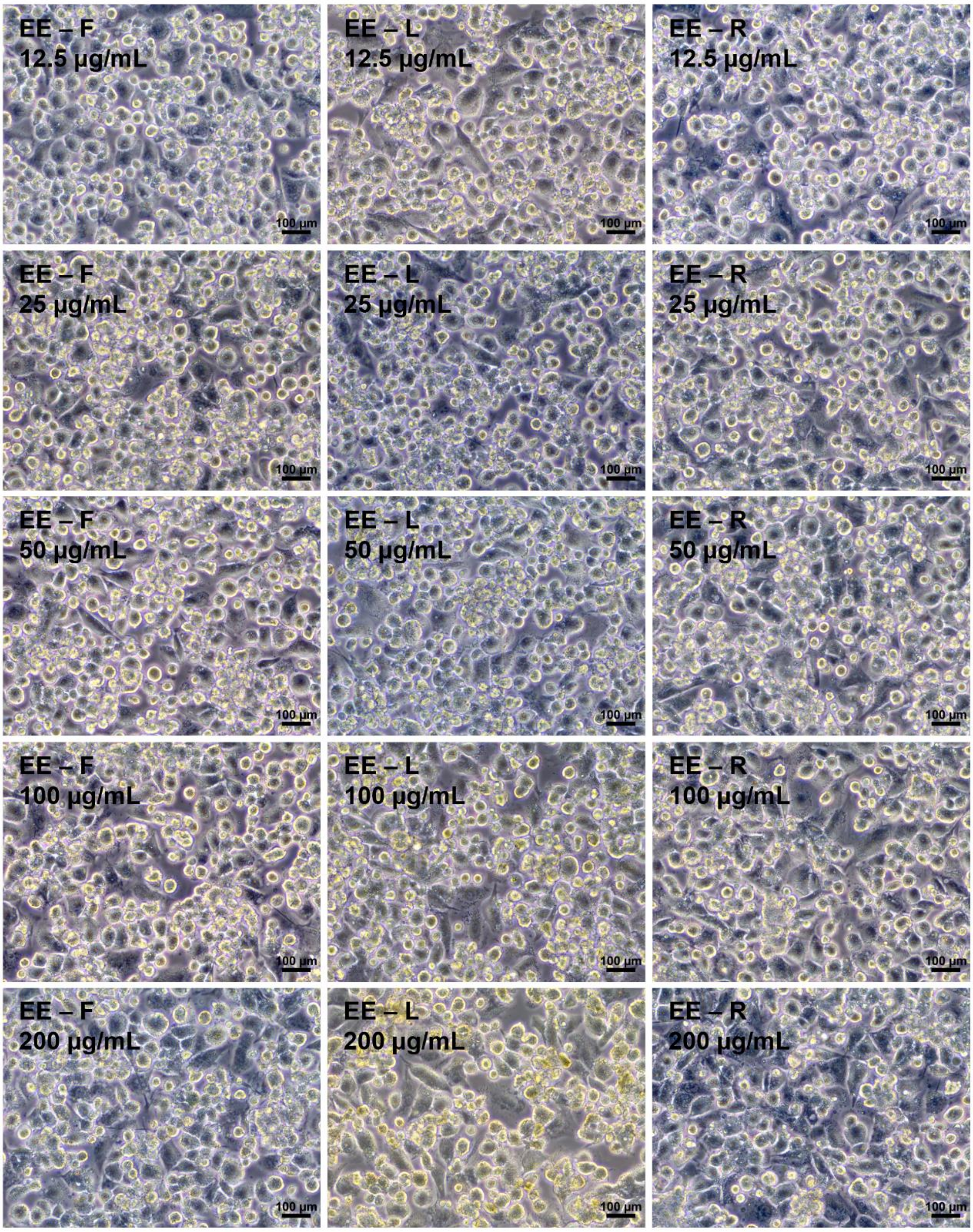

Figure S3. Optical micrographs of non-stimulated macrophages cultured in the presence of ethanolic extracts (EE; 12.5 - 200 µg/mL) obtained from *E. purpurea* flowers (F), leaves (L), and roots (R) cultured for 24 h. Scale bar = 100 µm.

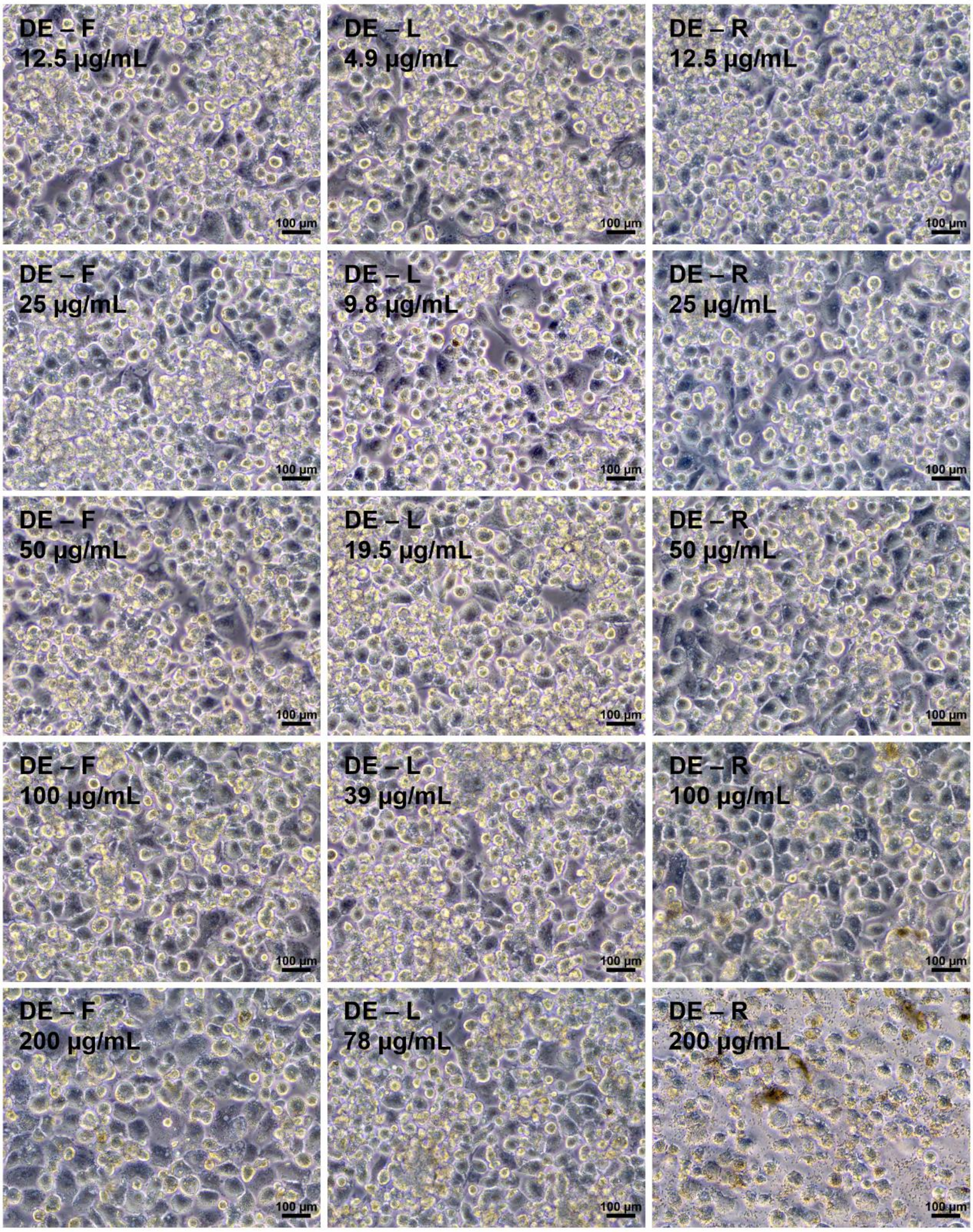

Figure S4. Optical micrographs of non-stimulated macrophages cultured in the presence of dichloromethanolic extracts (DE; 12.5 - 200 µg/mL) obtained from *E. purpurea* flowers (F), leaves (L), and roots (R) cultured for 24 h. Scale bar = 100 µm.

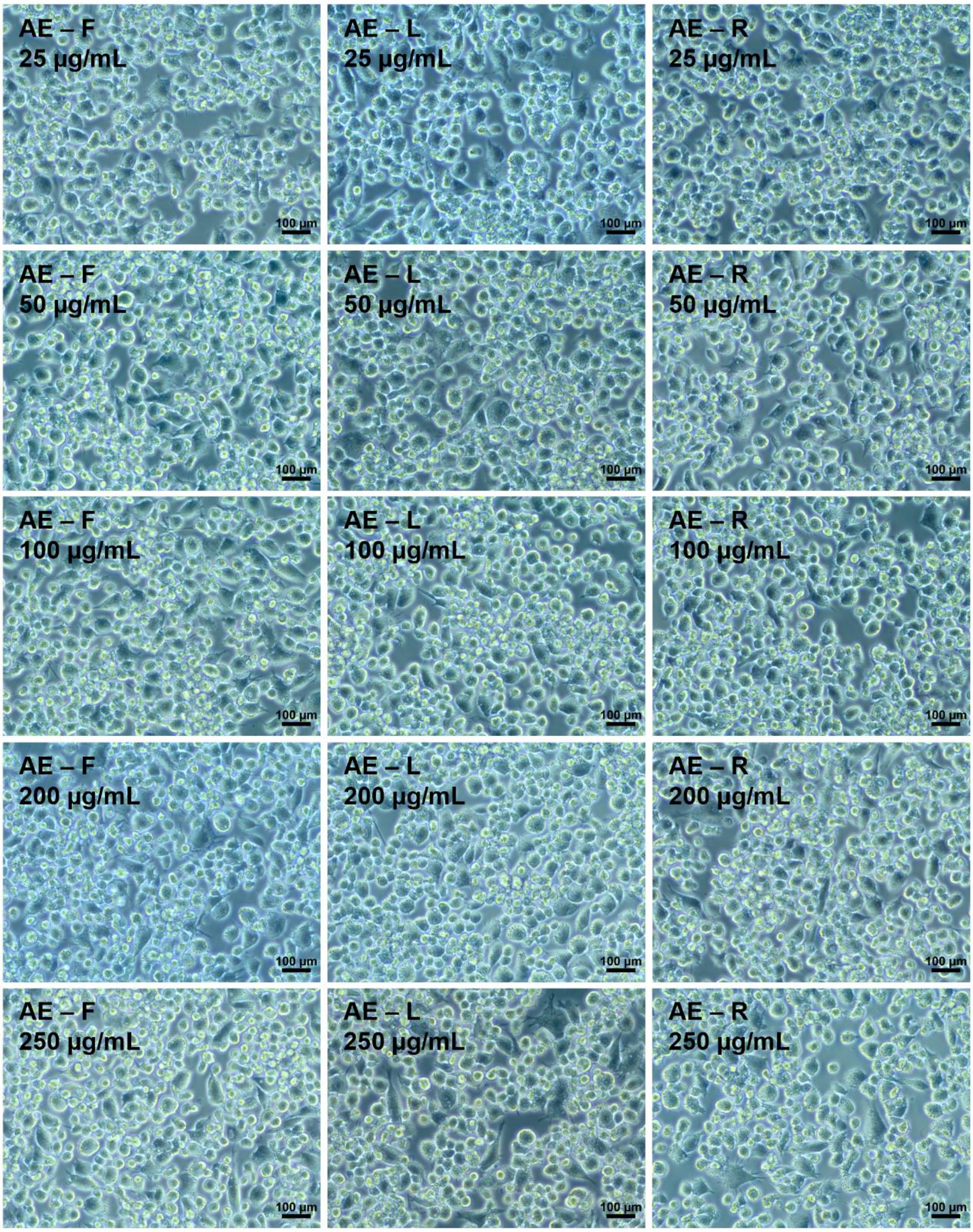

Figure S5. Optical micrographs of LPS-stimulated macrophages cultured in the presence of aqueous extracts (AE; 25 - 250 µg/mL) obtained from *E. purpurea* flowers (F), leaves (L), and roots (R) cultured for 24 h. Scale bar = 100 µm.

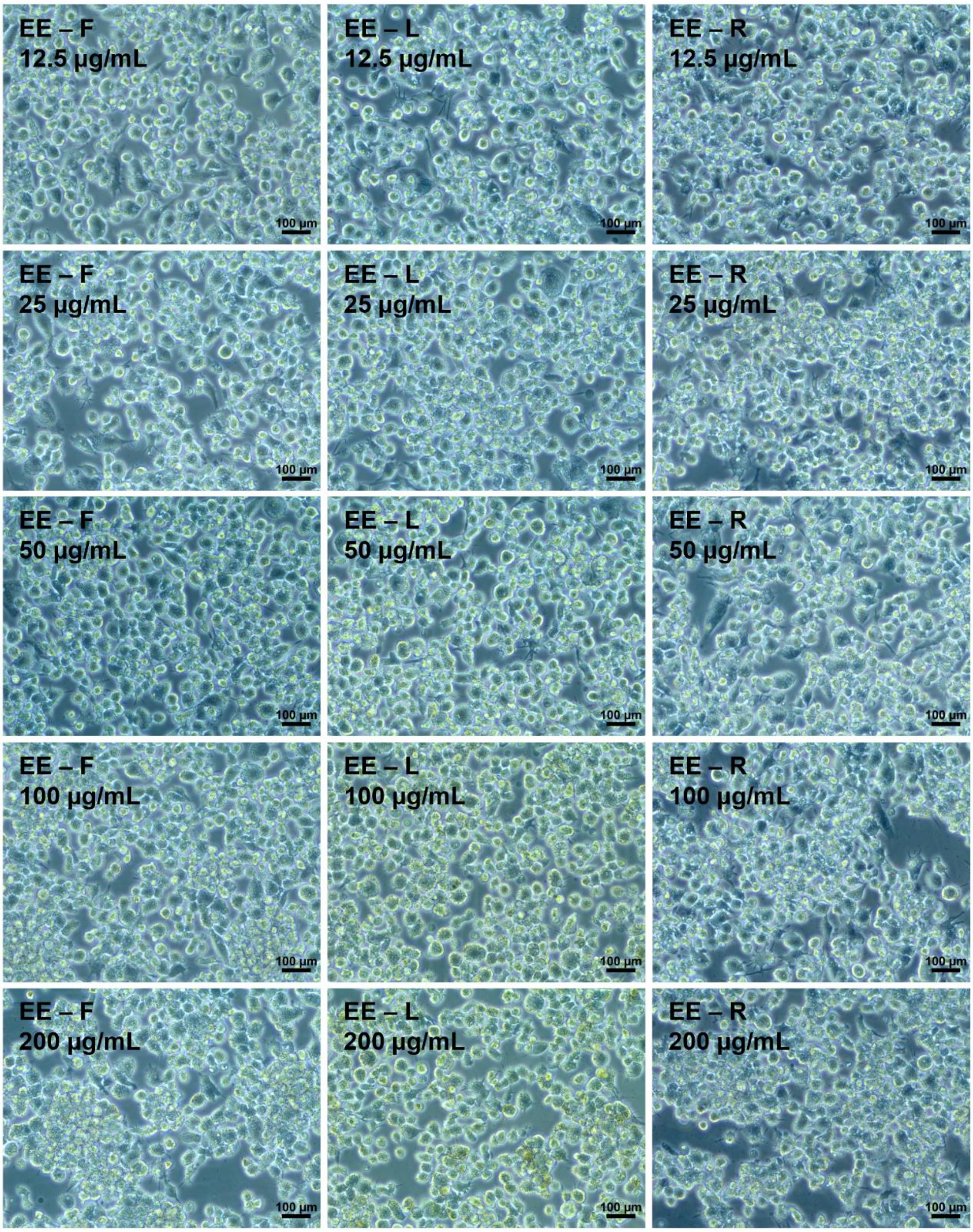

Figure S6. Optical micrographs of LPS-stimulated macrophages cultured in the presence of ethanolic extracts (EE; 12.5 - 200 μg/mL) obtained from *E. purpurea* flowers (F), leaves (L), and roots (R) cultured for 24 h. Scale bar = 100 μm.

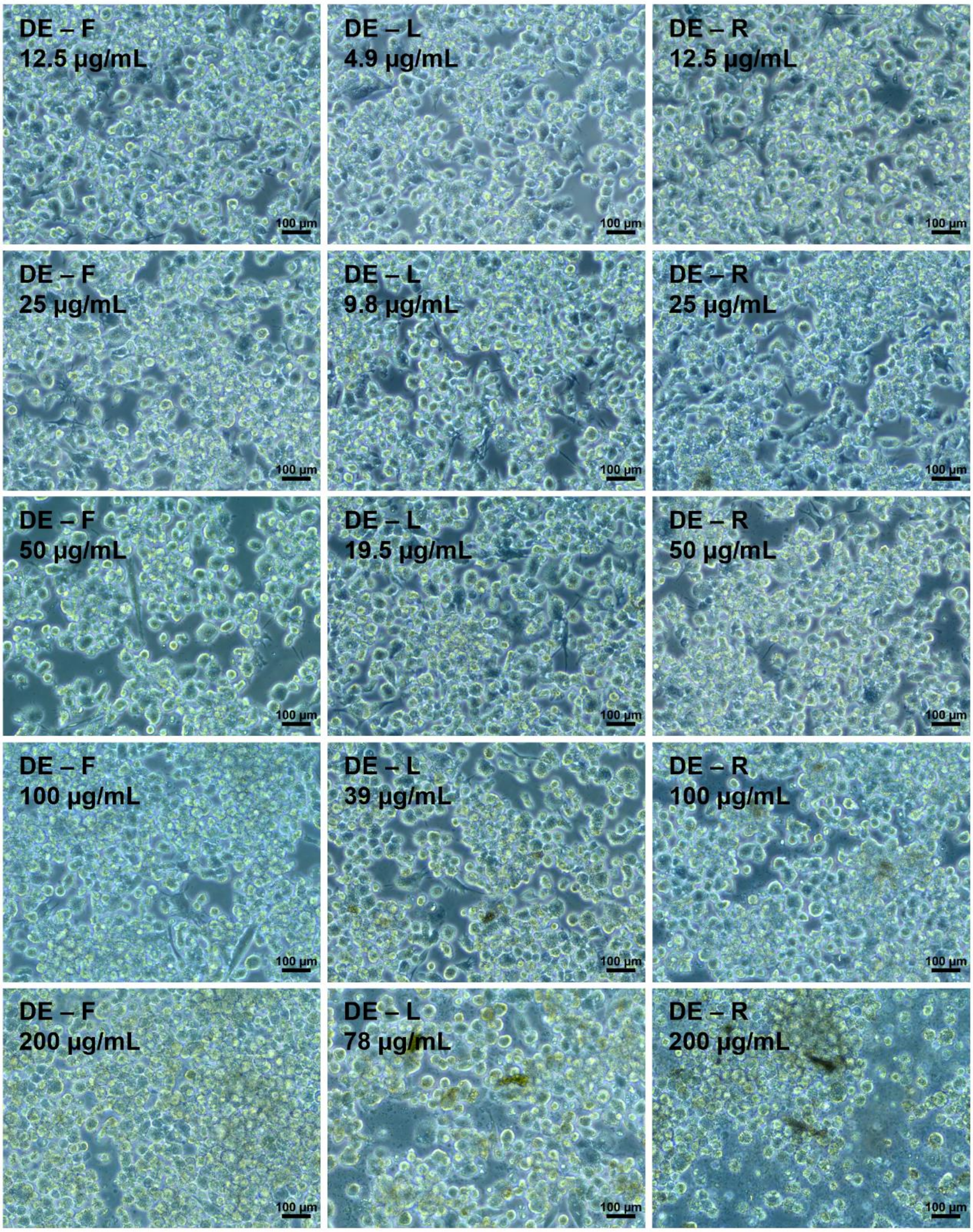

Figure S7. Optical micrographs of LPS-stimulated macrophages cultured in the presence of dichloromethanolic extracts (DE; 4.9 - 200 µg/mL) obtained from *E. purpurea* flowers (F), leaves (L), and roots (R) cultured for 24 h. Scale bar = 100 µm.

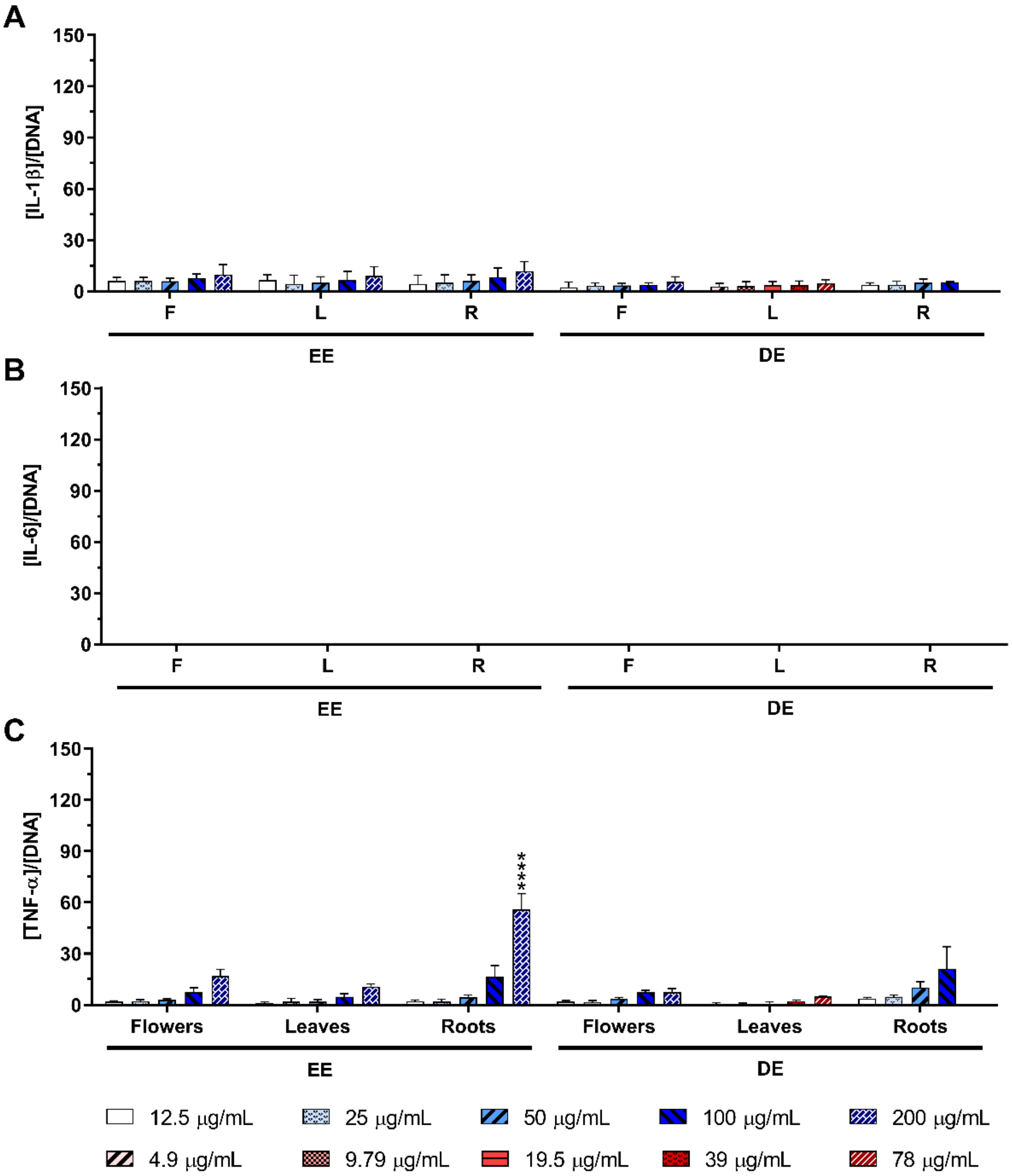

Figure S8. IL-1β (**A**), IL-6 (**B**), and TNF-*α* (**C**) production by non-stimulated macrophages cultured in the presence of different concentrations of the ethanolic (EE) and dichloromethanolic extract (DE) obtained from *E. purpurea* flowers (F), leaves (L), and roots (R) cultured for 24 h. Statistically significant differences are ** ($p < 0.0075$), *** ($p < 0.0006$), and **** ($p < 0.0001$) in comparison to the negative control (non-stimulated macrophages without treatment) for each different tested extract.

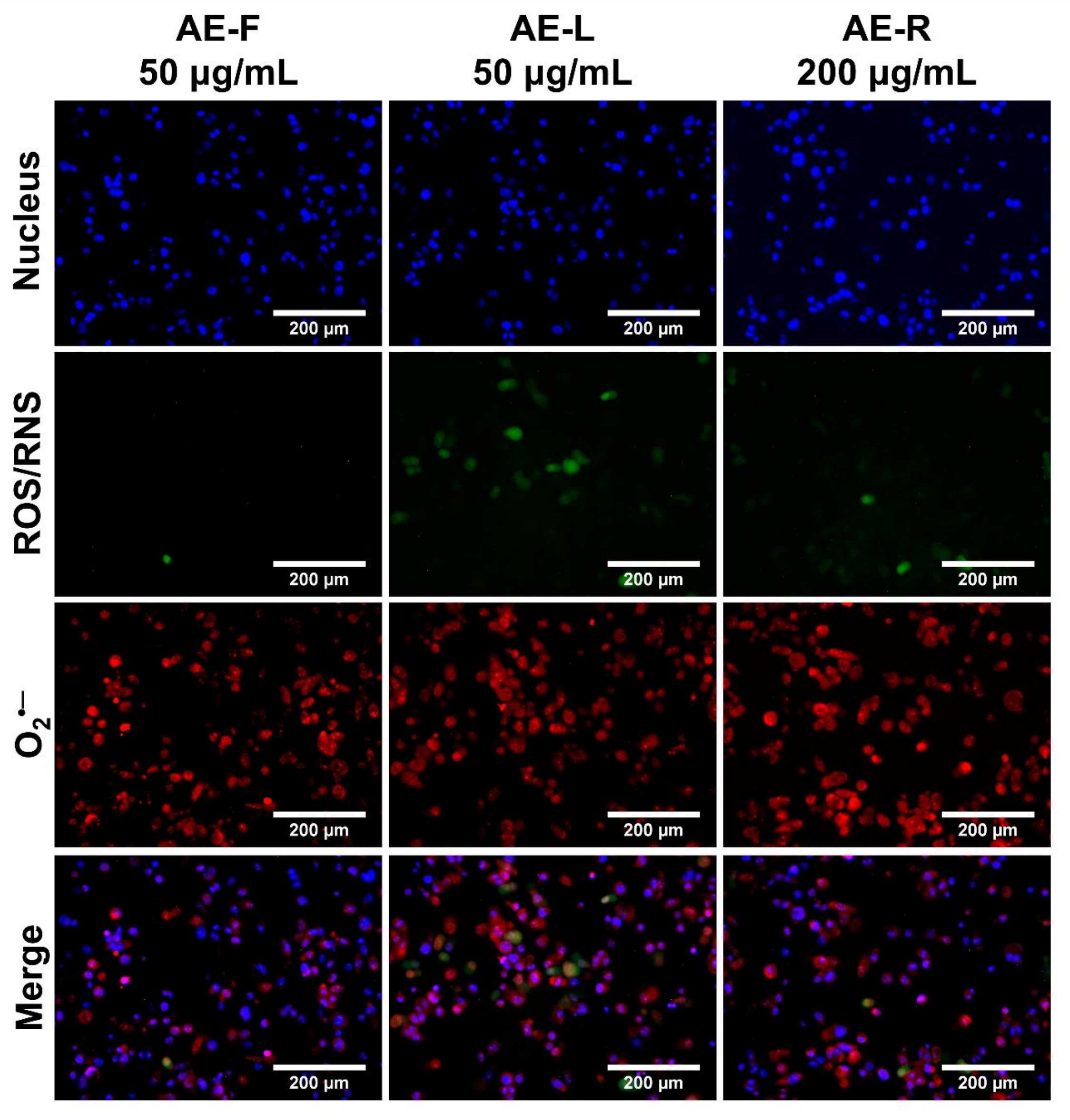

Figure S9. Intracellular ROS/RNS (green) and $O_2^{\bullet-}$ (red) production by non-stimulated macrophages (nucleus in blue) in the presence of aqueous extracts (AE; 50 or 200 µg/mL) obtained from flowers (F), leaves (L), and roots (R) of *E. purpurea* cultured for 24 h. Scale bar = 200 µm.

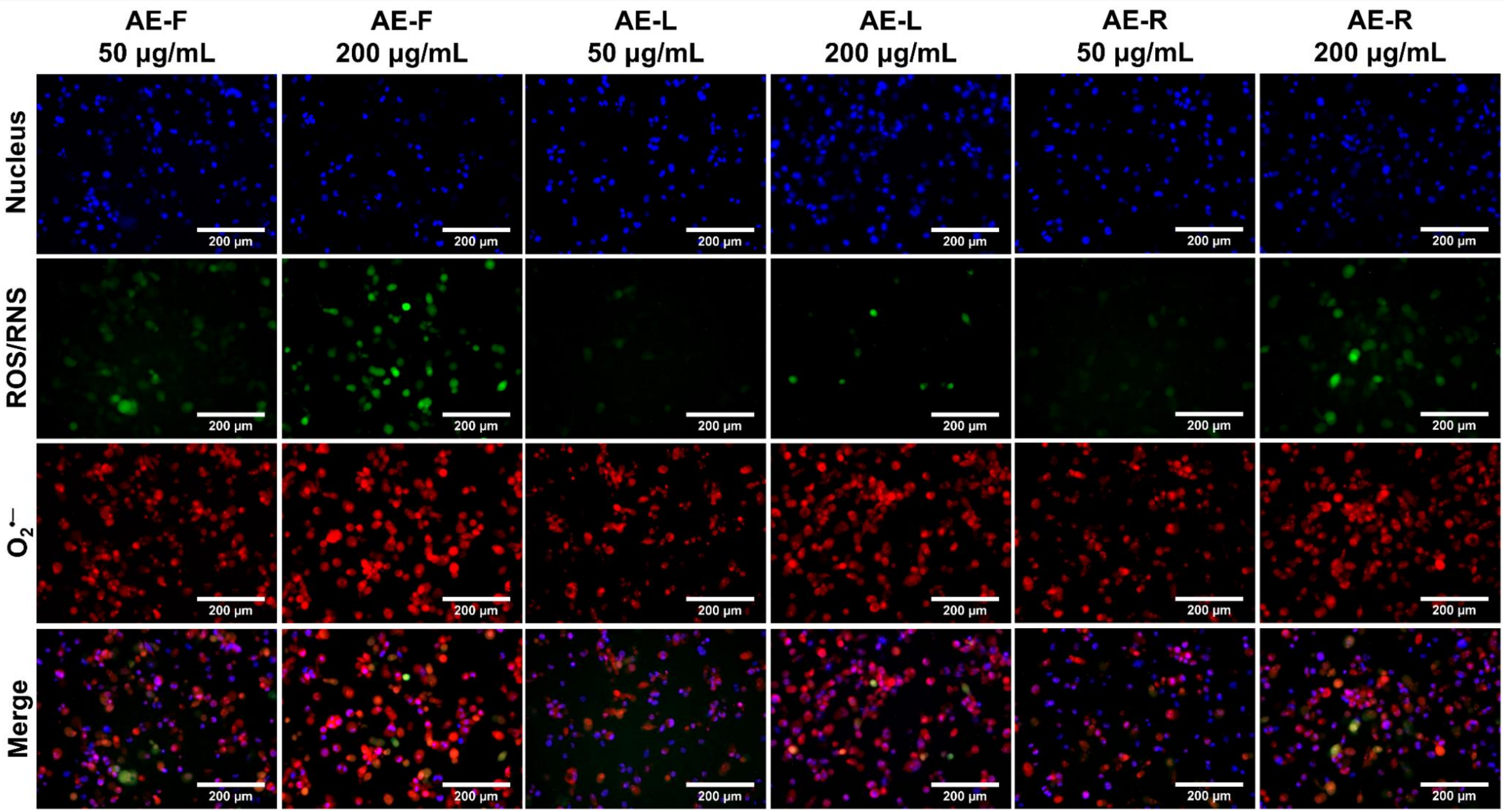

Figure S10. Intracellular ROS/RNS (green) and $O_2^{\bullet -}$ (red) production by LPS-stimulated macrophages in presence of aqueous extracts (AE; 50 and 200 µg/mL) obtained from flowers (F), leaves (L), and roots (R) of *E. purpurea* cultured for 24 h. Scale bar = 200 µm.

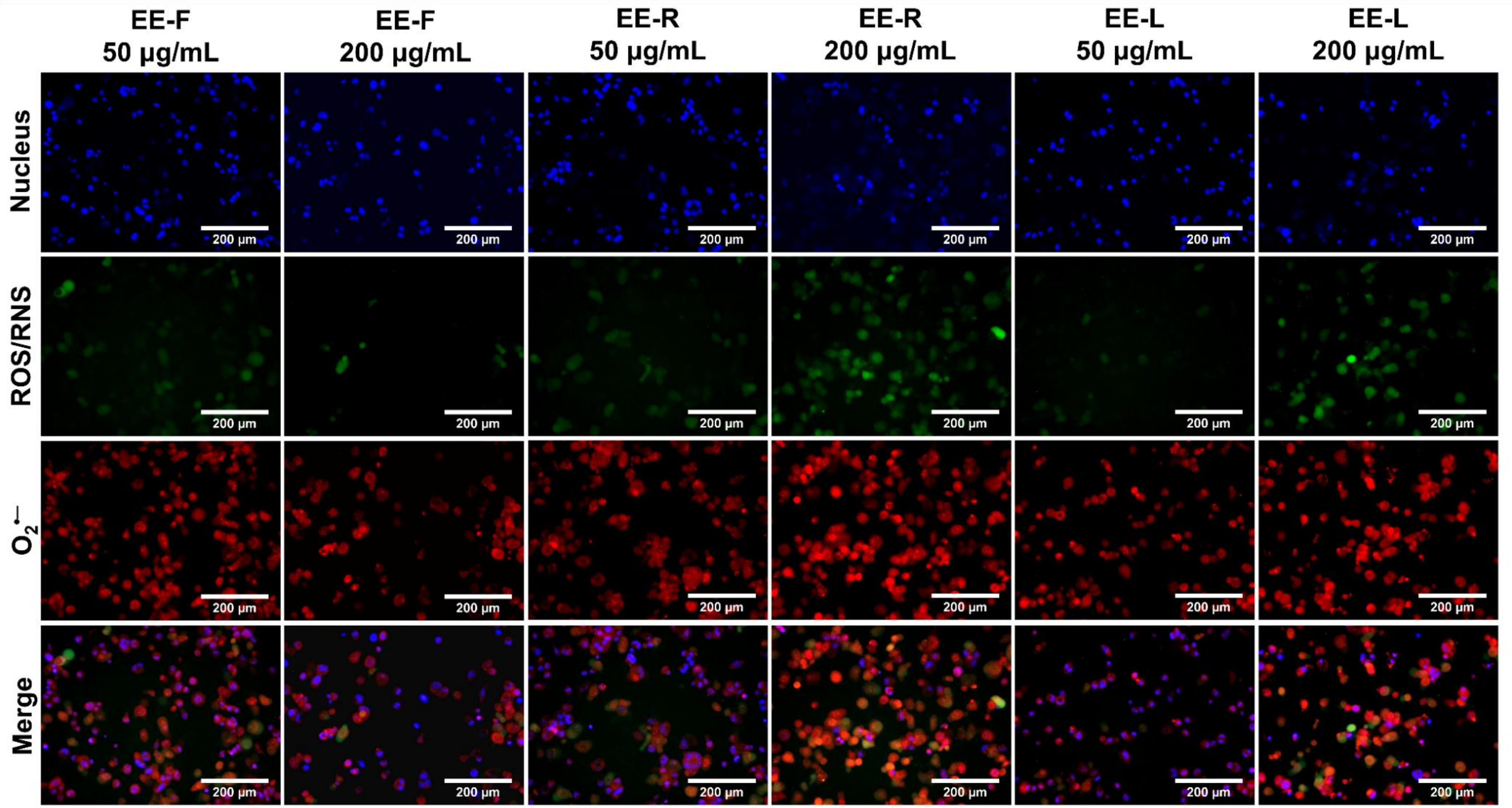

Figure S11. Intracellular ROS/RNS (green) and $O_2^{\bullet-}$ (red) production by LPS-stimulated macrophages in presence of ethanolic extracts (EE; 50 and 200 µg/mL) obtained from flowers (F), leaves (L), and roots (R) of *E. purpurea* cultured for 24 h. Scale bar = 200 µm.

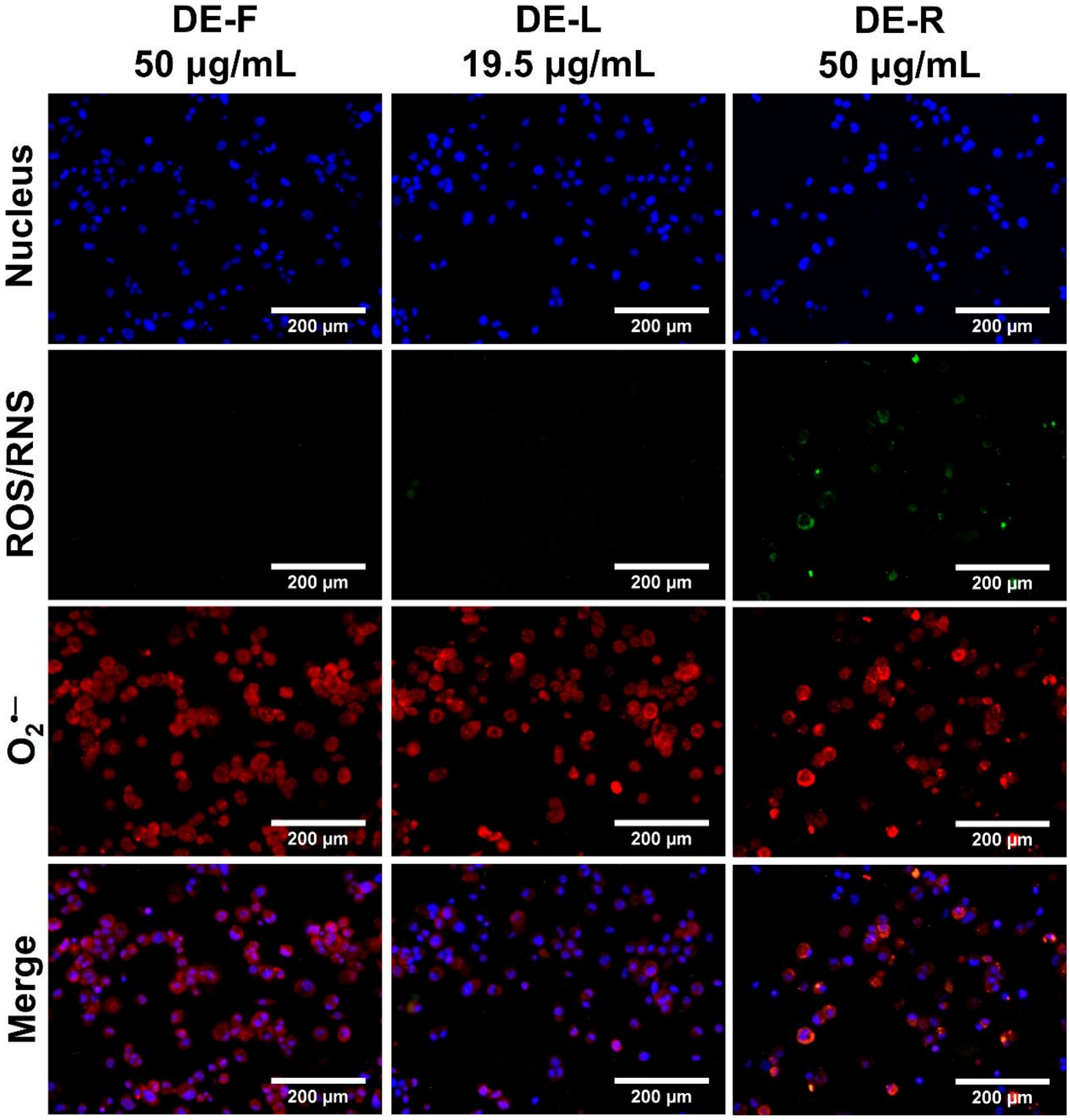

Figure S12. Intracellular ROS/RNS (green) and $O_2^{\bullet -}$ (red) production by LPS-stimulated macrophages in presence of dichloromethanolic extracts (DE; 50 and 200 µg/mL) obtained from flowers (F), leaves (L), and roots (R) of *E. purpurea* cultured for 24 h. Scale bar = 200 µm.

References

1. Cech, N.B.; Eleazer, M.S.; Shoffner, L.T.; Crosswhite, M.R.; Davis, A.C.; Mortenson, A.M. High performance liquid chromatography/electrospray ionization mass spectrometry for simultaneous analysis of alkamides and caffeic acid derivatives from Echinacea purpurea extracts. J. Chromatogr. A 2006, 1103, 219–228, doi:https://doi.org/10.1016/j.chroma.2005.11.008.
2. Spelman, K.; Wetschler, M.H.; Cech, N.B. Comparison of alkylamide yield in ethanolic extracts prepared from fresh versus dry Echinacea purpurea utilizing HPLC–ESI-MS. J. Pharm. Biomed. Anal. 2009, 49, 1141–1149, doi:https://doi.org/10.1016/j.jpba.2009.02.011.
3. Pellati, F.; Epifano, F.; Contaldo, N.; Orlandini, G.; Cavicchi, L.; Genovese, S.; Bertelli, D.; Benvenuti, S.; Curini, M.; Bertaccini, A.; et al. Chromatographic Methods for Metabolite Profiling of Virus- and Phytoplasma-Infected Plants of Echinacea purpurea. J. Agric. Food Chem. 2011, 59, 10425–10434, doi:10.1021/jf2025677.
4. Thomsen, M.O.; Fretté, X.C.; Christensen, K.B.; Christensen, L.P.; Grevsen, K. Seasonal Variations in the Concentrations of Lipophilic Compounds and Phenolic Acids in the Roots of Echinacea purpurea and Echinacea pallida. J. Agric. Food Chem. 2012, 60, 12131–12141, doi:10.1021/jf303292t.
5. He, X.; Lin, L.; Bernart, M.W.; Lian, L. Analysis of alkamides in roots and achenes of Echinacea purpurea by liquid chromatography–electrospray mass spectrometry. J. Chromatogr. A 1998, 815, 205–211, doi:https://doi.org/10.1016/S0021-9673(98)00447-6.

6. Mudge, E.; Lopes-Lutz, D.; Brown, P.; Schieber, A. Analysis of alkylamides in Echinacea plant materials and dietary supplements by ultrafast liquid chromatography with diode array and mass spectrometric detection. J. Agric. Food Chem. 2011, 59, 8086–8094, doi:10.1021/jf201158k.
7. Bauer, R.; Remiger, P. TLC and HPLC Analysis of Alkamides in Echinacea Drugs1,2. Planta Med. 1989, 55, 367–371, doi:10.1055/s-2006-962030.
